# Using genetic buffering relationships identified in fission yeast to reveal susceptibilities in cells lacking hamartin or tuberin function

**DOI:** 10.1242/bio.031302

**Published:** 2017-12-20

**Authors:** Ashyad Rayhan, Adam Faller, Ryan Chevalier, Alannah Mattice, Jim Karagiannis

**Affiliations:** Department of Biology, The University of Western Ontario, London, ON N6A-5B7, Canada

**Keywords:** Tuberous sclerosis complex, *Schizosaccharomyces pombe*, mTOR, Genetic buffering

## Abstract

Tuberous sclerosis complex is an autosomal dominant disorder characterized by benign tumors arising from the abnormal activation of mTOR signaling in cells lacking TSC1 (hamartin) or TSC2 (tuberin) activity. To expand the genetic framework surrounding this group of growth regulators, we utilized the model eukaryote *Schizosaccharomyces pombe* to uncover and characterize genes that buffer the phenotypic effects of mutations in the orthologous *tsc1* or *tsc2* loci. Our study identified two genes: *fft3* (encoding a DNA helicase) and *ypa1* (encoding a peptidyle-prolyl cis/trans isomerase). While the deletion of *fft3* or *ypa1* has little effect in wild-type fission yeast cells, their loss in *tsc1Δ* or *tsc2Δ* backgrounds results in severe growth inhibition. These data suggest that the inhibition of Ypa1p or Fft3p might represent an ‘Achilles’ heel’ of cells defective in hamartin/tuberin function. Furthermore, we demonstrate that the interaction between *tsc1*/*tsc2* and *ypa1* can be rescued through treatment with the mTOR inhibitor, torin-1, and that *ypa1Δ* cells are resistant to the glycolytic inhibitor, 2-deoxyglucose. This identifies *ypa1* as a novel upstream regulator of mTOR and suggests that the effects of *ypa1* loss, together with mTOR activation, combine to result in a cellular maladaptation in energy metabolism that is profoundly inhibitory to growth.

## Abstract (precis)

**Summary:** We exploit the BioGRID gene interaction database to identify and characterize susceptibilities in fission yeast cells lacking *tsc1* or *tsc2*, orthologs of the human *TSC1* and *TSC2* tumor suppressor genes.

## INTRODUCTION

Tuberous sclerosis complex (TSC) is an autosomal dominant disorder characterized by the development of benign tumors in tissues that include the heart, lung, kidney and brain. In addition, neurological phenotypes (e.g. seizures, learning disabilities, autism, hyperactivity, dementia and ataxia) are also commonly associated with the disorder. TSC affects as many as 40,000 individuals in the USA and between 1 to 2 million individuals worldwide. It has an estimated prevalence of one in 6000-8000 newborns and is seen in all races and in both sexes (Henske et al., 2016; Randle, 2017; Rosset et al., 2017). There is no cure.

TSC is caused by loss-of-function mutations in either *TSC1* (encoding the protein, hamartin) or *TSC2* (encoding the protein, tuberin). These genes function, at least in part, to inhibit the mechanistic target of rapamycin (mTOR) signaling pathway, which serves as a key regulator of cell proliferation, metabolism, and cell survival (Henske et al., 2016; Saxton and Sabatini, 2017). Orthologs of the *TSC1* and *TSC2* genes exist in a wide range of organisms, including the commonly used and genetically tractable model eukaryote, *Schizosaccharomyces pombe* (also known as fission yeast) (Hoffman et al., 2015).

While the inhibition of mTOR signaling through rapamycin treatment has shown some therapeutic promise, rapamycin-independent pathways related to ‘non-canonical’ *TSC1* and *TSC2* functions are also likely to play a role with respect to the manifestation of the disorder (Neuman and Henske, 2011). In addition, while rapamycin treatment alone has shown some efficacy in limiting the growth of tumor cells, combination therapies involving rapamycin and secondary drugs have proven even more useful in treating certain cancers (Li et al., 2014). Lastly, it is also clear that rapamycin treatment is purely cytostatic. Thus, the identification of alternate ‘drugable’ targets (the inhibition of which might result in a cytocidal response) may provide more effective options for treatment (Medvetz et al., 2015; Neuman and Henske, 2011; Switon et al., 2016).

Fortunately, the relatively recent development of genetic interaction analysis has provided a novel and unbiased avenue with which to pursue this goal. In such assays the phenotypic effects of a ‘query’ mutation – both alone and in combination with a panel of mutations representing the remaining genes in the genome – are assayed to uncover both negative and positive genetic interactions on a genome-wide scale (Baryshnikova et al., 2013; Dixon et al., 2009b; Kuzmin et al., 2016). In this way, functional relationships between the query gene and all other genes in the genome can be characterized in a systematic and unbiased manner. Such strategies have been used successfully in a wide variety of model systems including *Saccharomyces cerevisiae*, *S. pombe*, *Escherichia* *coli*, *Caenorhabditis* *elegans*, *Drosophila* *melanogaster*, and more recently in mammalian cell lines (Butland et al., 2008; Byrne et al., 2007; Costanzo et al., 2016; Housden et al., 2015, 2017; Ryan et al., 2012; Shen et al., 2017).

Of particular relevance to TSC are genes that negatively interact with both *TSC1* and *TSC2*. Given the fact that tumor formation arises from loss of heterozygosity, this characteristic identifies such genes as potential therapeutic targets. This is to say, drugs inhibiting a negative interactor would presumably suppress only the growth of tumor cells (which bear two mutant copies of the affected TSC gene: the inherited mutant germline copy, and the copy affected by the ‘second-hit’) while leaving phenotypically normal cells (carrying only the mutant germline copy) unaffected. In addition, the fact that these interacting genes ‘buffer’ the phenotypic effects of hamartin and tuberin loss-of-function mutations, suggests that their detailed molecular/genetic analysis might provide novel insight into the molecular pathology of TSC and its many diverse associated phenotypes.

Due to the paucity of genome-wide genetic interaction data in human cell lines, we have explored the genetic framework surrounding the fission yeast orthologs of human *TSC1* and *TSC2* in the hope of identifying susceptibilities generally conserved in cells deficient in hamartin or tuberin function. While limited data exist, current research does indeed indicate some conservation in genetic interaction networks between organisms separated by significant evolutionary distances (Dixon et al., 2008, 2009a; Tosti et al., 2014).

In this report, we present the results of this preliminary exploration and identify *fft3* (encoding a SMARCAD1 family ATP-dependent DNA helicase) and *ypa1* (encoding a peptidyl-prolyl cis-trans isomerase) as strong negative interactors. While deletion of either gene has little phenotypic effect in normal cells, their loss in either *tsc1* or *tsc2* gene deletion mutants profoundly inhibits growth. Furthermore, we show that the targeted loss of Fft3p ATPase activity confers a similar growth defect in *tsc1Δ* or *tsc2Δ* mutant backgrounds. These data suggest that the inhibition of either Ypa1p or Fft3p (through targeting the ATPase domain) might represent an ‘Achilles’ heel’ of cells defective in hamartin or tuberin function. In addition, we identify Ypa1p as a novel upstream regulator of mTOR and present evidence suggesting that the phenotypic effects of *ypa1* loss, together with ectopic mTOR activation, combine to induce a cellular maladaptation in energy metabolism that is profoundly inhibitory to growth.

## RESULTS

### Inspection of the *S. pombe* genetic interaction network reveals a group of nine interactors common to both *tsc1* and *tsc2*

Genes that negatively interact with both *TSC1* and *TSC2* represent potential therapeutic targets (i.e. drugs inhibiting a negative interactor would be predicted to suppress the growth of tumor cells while leaving phenotypically normal cells unaffected). Due to the paucity of genetic interaction data in human cell lines, together with the abundance of such data in *S. pombe*, we initiated an exploratory study examining the genetic interaction network encompassing the fission yeast orthologs of human *TSC1* and *TSC2*.

We began by definitively identifying the orthologous *S. pombe* genes using the DRSC Integrative Ortholog Prediction Tool. This revealed two open reading frames, SPAC22F3.13, and SPAC630.13c, as the sole orthologs of *TSC1* and *TSC2*, respectively (Supplementary Files S1 and S2). Aptly named *tsc1* and *tsc2*, these genes were previously identified as sharing significant similarity with human *TSC1* and *TSC2* and have been extensively characterized with respect to nutrient uptake and their roles in regulating the fission yeast Tor1p and Tor2p proteins (Aspuria and Tamanoi, 2008; Matsumoto et al., 2002; Nakase et al., 2013; van Slegtenhorst et al., 2004, 2005; Weisman et al., 2005, 2007). Fission yeast Tsc1p and Tsc2p interact physically, just like their human counterparts (Matsumoto et al., 2002), and share a similar overall domain structure with their respective human orthologs (Fig. S1).

Next, we mined the publicly available BioGRID database to identify genes exhibiting interactions with either *tsc1* or *tsc2*. BioGRID is an open access database that catalogs protein, genetic and chemical interactions in all commonly used model organisms as well as in humans. Annotated from over 48,000 publications, this resource provides the most comprehensive archive of interactions currently available (Chatr-Aryamontri et al., 2017; Stark et al., 2006).

**Fig. 1.**
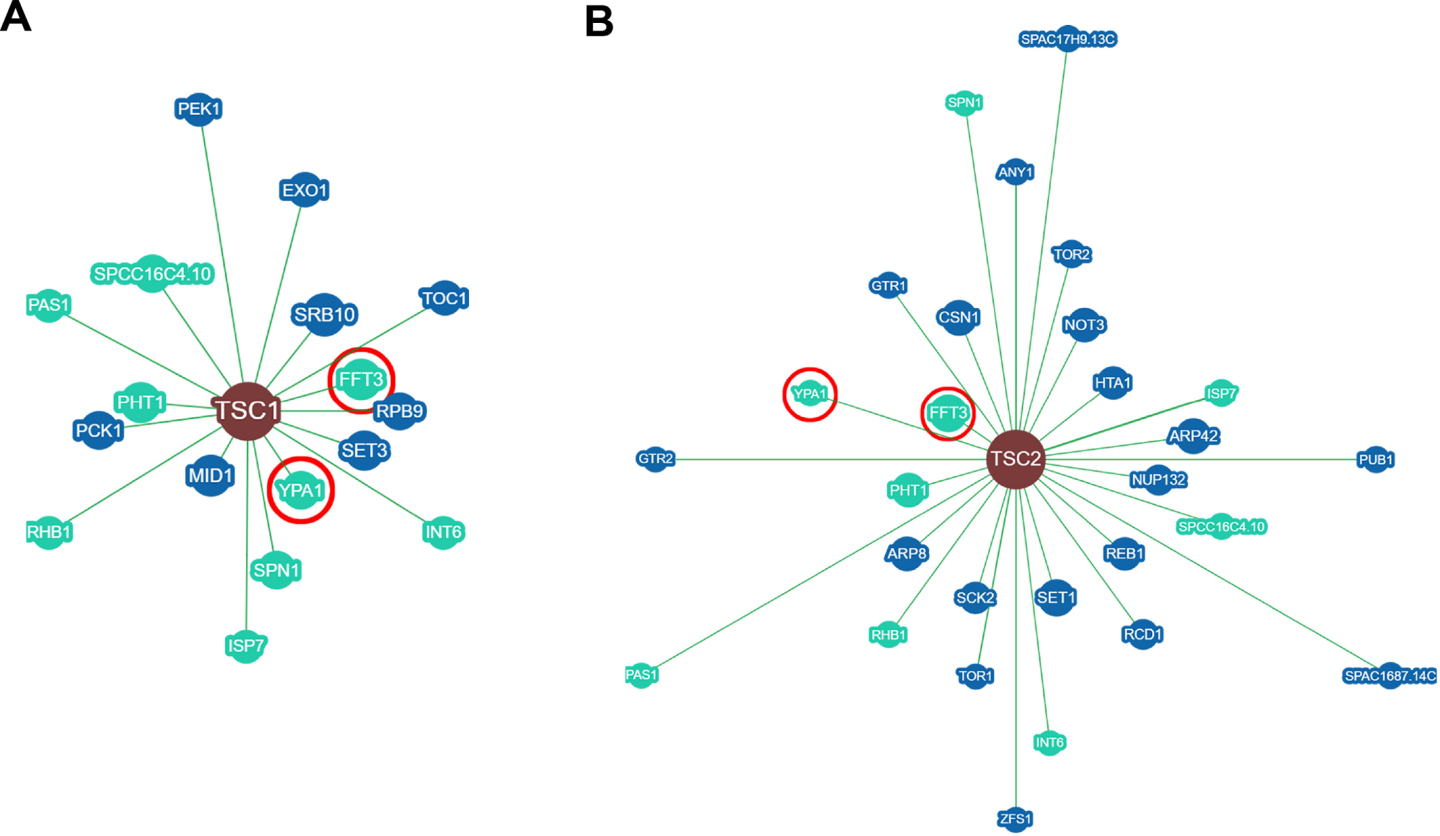
**Network diagram displaying fission yeast genes that genetically interact with *tsc1* or *tsc2*.** The diagram was created using the embedded BioGRID webtool powered by CytoscapeJS (http://thebiogrid.org/). Genes common to both *tsc1* (A) and *tsc2* (B) are shown in light blue. The *ypa1* and *fft3* genes are encircled in red.

This analysis revealed a total of 99 *tsc1*-specific, and 143 *tsc2*-specific genetic interactions. These data were filtered by removing interactions that were supported by only a single observation (i.e. the interactions of the filtered set are supported by two or more experimental results). The remaining 17 *tsc1*-specific and 28 *tsc2*-specific interactions were then filtered again by removing genes that interact with *tsc1*, but not *tsc2*, as well as genes that interact with *tsc2*, but not *tsc1*. Of the remaining nine genes, two (*fft3* and *ypa1*) were chosen for further detailed analysis as they display strong negative interactivity with *tsc1* and *tsc2*, and they themselves possess clear human orthologs (Fig. 1). The *fft3* gene encodes a SMARCAD1 family ATP-dependent DNA helicase that is known to suppress nucleosome turnover and that is involved in controlling nuclear organization and chromatin structure (Lee et al., 2017; Steglich et al., 2015; Strålfors et al., 2011; Taneja et al., 2017). The *ypa1* gene encodes a peptidyl-prolyl cis/trans-isomerase involved in the regulation of protein phosphatase 2A (PP2A) and PP2A-like enzymes (Goyal and Simanis, 2012; Guo et al., 2014; Jordens et al., 2006; Leulliot et al., 2006). Neither has been previously characterized with respect to their relationship to TSC or mTOR signaling in any system.

### Validation and quantitation of the identified negative genetic interactions

We began the next phase of the study by first confirming and carefully quantitating the severity of the observed negative genetic interactions. To this end, we first created *tsc1* and *tsc2* knockout strains in which the *ura4^+^* deletion cassette was flanked by *loxP* and *loxM3* sites. These sites were added so that we could later introduce alternate *tsc1/tsc2* alleles using a technique based on recombinase mediated cassette exchange (see Materials and Methods). To confirm that the constructed knockout strains were true loss-of-function variants, we grew the mutants on EMM plates supplemented with 60 µg/ml canavanine (a toxic arginine analog). As expected, the knockout strains, in contrast to a wild-type control, were able to proliferate on canavanine media, reflecting the inability of *tsc1* or *tsc2* mutants to properly transport amino acids from the extracellular environment into the cell (van Slegtenhorst et al., 2004) (Fig. S2).

**Fig. 2.**
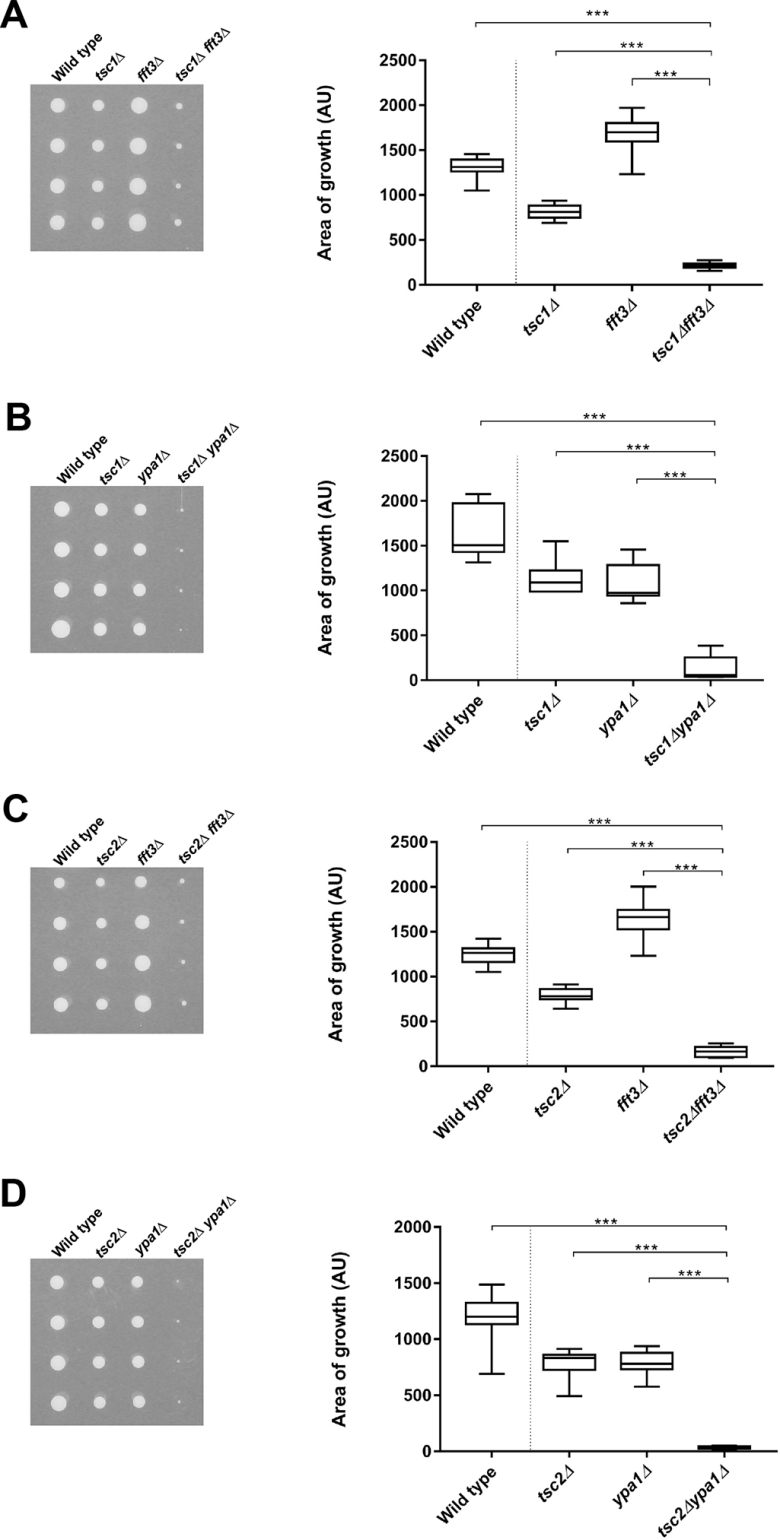
**Cells bearing gene deletions in either *fft3* or *ypa1* are profoundly inhibited for growth in *tsc1/tsc2* mutant backgrounds.** (A,C) *fft3*. (B,D) *ypa1*. Individual cells of the indicated genotype were seeded (in quadruplicate) onto YES plates. Photographs (left) show colony size after 3 days' growth. Box and whisker plots (right) describe colony size (arbitrary units) after 3 days' growth on YES medium at 30°C. ***P<0.001.

Having validated that the knockout strains were true loss-of-function mutants, we individually crossed the *tsc1* and *tsc2* gene deletion strains to *fft3Δ* or *ypa1Δ* mutants (obtained as part of version 4 of the Bioneer *S. pombe* gene deletion collection) (Kim et al., 2010). Double mutants were recovered and assayed for growth alongside a wild-type control and the respective single mutant strains. Assays were performed by monitoring colony size in rich media at 30°C over 5 days (see Materials and Methods). To avoid the possible confounding effects of background auxotrophic mutations, all strains examined in this way were prototrophic. As shown in Fig. 2, the colony sizes of *fft3Δ tsc1Δ*, *fft3Δ tsc2Δ*, *ypa1Δ tsc1Δ* and *ypa1Δ tsc2Δ* double mutants were significantly reduced (*P*<0.05) in comparison to both the wild-type control and the respective single mutants.

In order to quantitate the severity of the interactions, we next calculated ε-values (for each double mutant) corresponding to each of the four commonly used models of neutrality: the Product, Min, Log, and Additive models (Mani et al., 2008). These values provide a measure of the deviation of the observed double mutant fitness from the fitness expected if there was a ‘neutral’ interaction (see Materials and Methods). As shown in Table 1, the calculated ε-values were less than zero for each of the neutrality models, indicating that the double mutant phenotypes were indeed more severe than expected based on the phenotypes of the individual single mutants.

**Table 1.**
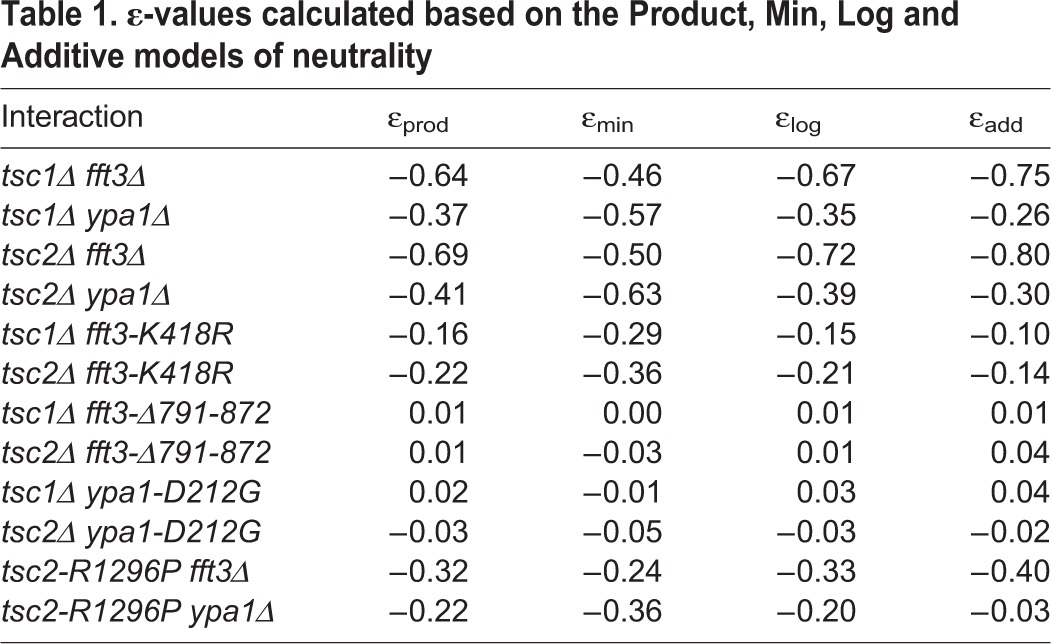
**ε-values calculated based on the Product, Min, Log and Additive models of neutrality**

### Neighboring gene effects

Importantly, recent work in *S. cerevisiae* has shown that negative interactions can be falsely annotated due to neighboring gene effects (NGEs). In such cases, the deletion of a given gene affects the proper functioning of an adjacent gene leading to improper annotation of negative interactions (Atias et al., 2016; Baryshnikova and Andrews, 2012; Ben-Shitrit et al., 2012). While the mechanism of these effects remains unknown, it is possible that the deletion of a given locus alters chromatin structure and/or the regulatory characteristics of adjacent loci (Baryshnikova and Andrews, 2012). NGEs are relatively extensive (with measured rates ranging from 7% to 15%) and can distort large-scale genetic interactomes (Ben-Shitrit et al., 2012).

To eliminate the possibility that the loss-of-function of a gene adjacent to *fft3* or *ypa1* was in fact responsible for the observed negative interactions we examined the effects of *atg14*, *spac25a8.03c* and *lip2* gene deletions. *atg14* and *spac25a8.03c* are immediately adjacent to *fft3* while *lip2* is immediately downstream of *ypa1* (Fig. S3)*.* The gene immediately upstream of *ypa1*, *trm7*, is essential and thus could not be tested. The data obtained revealed that the *tsc1Δ atg14Δ*, *tsc1Δ spac25a8.03cΔ*, *tsc1Δ lip2Δ*, *tsc2Δ atg14Δ*, *tsc2Δ spac25a8.03cΔ* and *tsc2Δ lip2Δ* double mutants did not exhibit significant growth defects relative to the relevant single mutants (data not shown). While difficult to rule out NGEs resulting from the combined perturbation of both the gene of interest and a neighboring gene, these results at minimum demonstrate that the synthetic interactions characterized in this study are not influenced by NGEs resulting from the simple loss-of-function of adjacent genes.

### *fft3* mutants defective in ATPase activity negatively interact with both *tsc1Δ* and *tsc2Δ*

**Fig. 3.**
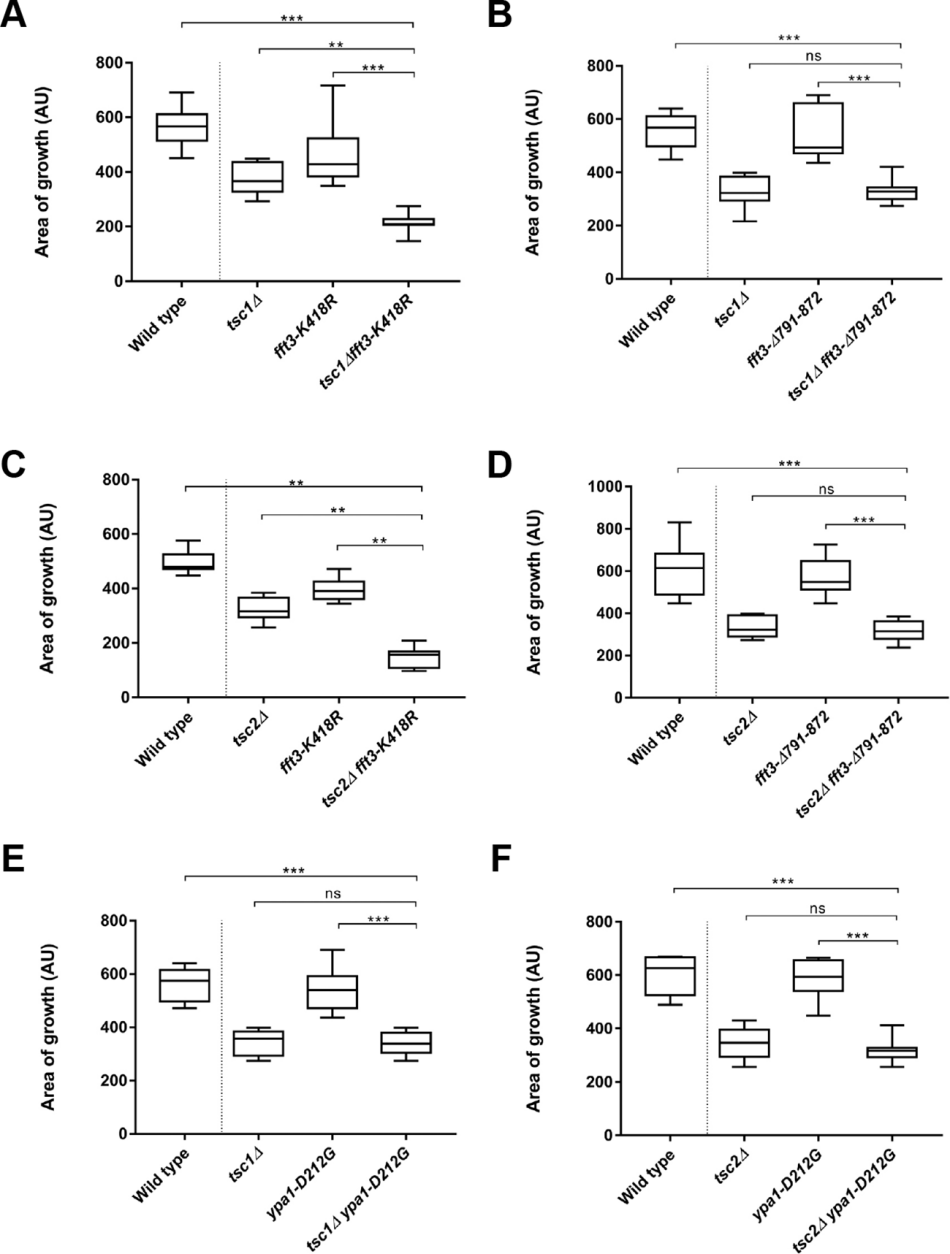
**Cells bearing the *fft3-K418R* mutation are inhibited for growth in *tsc1/tsc2* mutant backgrounds, while cells bearing the *fft3-Δ791-872* mutation, or the *ypa1-D212G* mutation, show no synthetic growth defects with *tsc1/tsc2* gene deletions.** (A,C) *fft3-K418R mutation*. (B,D) *fft3-Δ791-872* mutation. (E,F) *ypa1-D212G mutation*. Individual cells of the indicated genotype were seeded (in quadruplicate) onto YES plates. Box and whisker plots describe colony size (arbitrary units) after 3 days' growth on YES medium at 30°C. **P<0.01; ***P<0.001; ns, not significant.

We were next interested in identifying specific domains in Fft3p or Ypa1p that might be pharmacologically targeted (in *tsc1* or *tsc2* mutant backgrounds) to recapitulate a negative interaction. Since Fft3p exhibits both helicase and ATPase activity, it was unclear if one or both biochemical functions needed to be abrogated in order to confer a negative interaction. We thus created two strains: one expressing an *fft3* allele bearing a K418R substitution (ATPase dead) and one where the helicase domain (residues 791-872) was completely deleted (Fig. S4) (Costelloe et al., 2012). These strains were individually crossed to *tsc1Δ* and *tsc2Δ* knockouts and the double mutants isolated. Interestingly, a negative interaction was observed in *fft3-K418R tsc1Δ* and *fft3-K418R tsc2Δ* double mutants, but not in *fft3-Δ791-872 tsc1Δ* or *fft3-Δ791-872 tsc2Δ* mutants (Fig. 3A-D, Table 1). This suggests that the pharmacological inhibition of the ATPase domain of Fft3p might be specifically inhibitory to the growth of cells deficient in hamartin or tuberin function.

Using a similar line of reasoning, we also constructed single and double mutant strains expressing a *ypa1* allele (*ypa1-D212G*) that was reported to abrogate prolyl isomerase (PPIase) activity (Fig. S5) (Leulliot et al., 2006). Interestingly, in this case no negative interaction was observed (Fig. 3E,F, Table 1). This indicates that the loss of a yet unidentified molecular function is in fact responsible for the negative genetic interactions observed between *ypa1Δ* and *tsc1Δ/tsc2Δ*.

### Using recombinase mediated cassette exchange to examine alternate *tsc1* or *tsc2* alleles

We were next interested in establishing an *S. pombe* platform where we could quickly and easily construct strains expressing wild-type or mutant *tsc1* or *tsc2* alleles under the control of their native promoters. To this end, we employed a previously described system based on recombinase-mediated cassette exchange (RMCE) (Watson et al., 2008). As we had previously introduced *loxP* and *loxM3* sites into the respective deletion cassettes, we could now replace the *ura4^+^* selectable marker present in the *tsc1::ura4^+^/tsc2::ura4^+^* knockout strains (hereafter referred to as the ‘base’ strains) by simply transforming the respective knockouts with a vector that expresses the Cre recombinase and that contains *tsc1* or *tsc2* sequence variants flanked by the identical *loxP* and *loxM3* sites (Fig. S6).

To test the RMCE system, we first exchanged the *ura4^+^* selectable markers present in the *tsc1::ura4^+^* and *tsc2::ura4^+^* base strains with wild-type *S. pombe tsc1* or *tsc2* (creating the *tsc1::tsc1^Sp^* and *tsc2::tsc2^Sp^* strains). As expected, cells of these genotypes were unable to proliferate on canavanine plates indicating that they were indeed expressing functional Tsc1p and Tsc2p (Fig. S2). In an attempt to create a system where we could study the human hamartin and tuberin proteins in yeast, we also asked if human *TSC1* or *TSC2* could substitute for the *S. pombe* versions of the genes. We thus used RMCE to exchange the *ura4^+^* selectable markers of the knockout strains with human *TSC1* or *TSC2* creating the *tsc1::TSC1^Hs^* and *tsc2::TSC2^Hs^* strains. Unfortunately, both strains (as well as *tsc1::TSC1^Hs^ tsc2::TSC2^Hs^* cells co-expressing both human TSC1 and TSC2) were able to proliferate on canavanine media. Thus, the human hamartin and tuberin proteins are unable to substitute for their fission yeast counterparts *in vivo* (Fig. S2).

Up to this point in the study, all of the genetic interaction analyses had been performed using complete deletions of *tsc1* or *tsc2*. We thus asked if the observed negative interactions were allele specific or if they might be observed more generally with all loss-of-function *tsc1* or *tsc2* alleles. To this end, we constructed Cre-recombinase exchange plasmids carrying *tsc1* or *tsc2* alleles bearing point mutations (*tsc1-L346H*, *tsc1-G432R*, *tsc2-G296E*, *tsc2-N1191K*, *tsc2-N1199S*, *tsc2-R1296P*) orthologous to those found clinically (Supplementary Files S1 and S2). These plasmids were transformed into the *tsc1::ura4^+^* or *tsc2::ura4^+^* base strains and cells expressing the respective *tsc1* or *tsc2* alleles were recovered (see Materials and Methods). To assess if the mutations did indeed result in loss-of-function, we assayed the growth of the *tsc1* or *tsc2* point mutant strains on canavanine media and observed that only two (*tsc2-N1191K*, *tsc2-R1296P*) were capable of growth (indicating that they alone represented true loss of function mutants) (data not shown). The two mutant alleles were then individually crossed into *fft3Δ* or *ypa1Δ* backgrounds to assess the growth rates of double mutant cells.

**Fig. 4.**
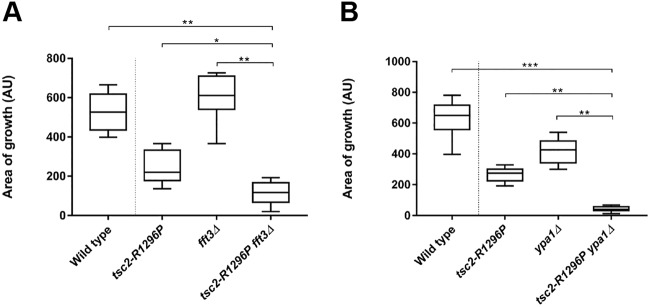
**Cells bearing the *tsc2-R1296P* mutation are inhibited for growth in *fft3* or *ypa1* mutant backgrounds.** (A) *fft3*. (B) *ypa1*. Individual cells of the indicated genotype were seeded (in quadruplicate) onto YES plates. Box and whisker plots describe colony size (arbitrary units) after 3 days' growth on YES medium at 30°C. *P<0.05; **P<0.01; ***P<0.001.

While both of the mutations clearly resulted in loss of *tsc2* function (based on the ability of the respective mutants to grow on canavanine plates) only one of the two alleles (*tsc2-R1296P*) displayed negative interactivity with both *fft3* and *ypa1* (Fig. 4, Table 1). In contrast, the *tsc2-N1191K* double mutants did not grow significantly slower than the respective single mutants (data not shown). Thus, the observed interactions between *tsc2* and *ypa1* or *fft3* are allele specific. This demonstrates that the loss of *fft3* or *ypa1* activity is not necessarily detrimental to growth in all cells exhibiting deficiencies in hamartin or tuberin function. Instead the results imply that genetic interaction might depend on the subtleties of Tsc1p/Tsc2p molecular function in an allele-specific manner (see Discussion).

### Treatment with the mTOR inhibitor, torin-1, rescues the negative genetic interaction observed between *ypa1* and *tsc1/tsc2*, but not between *fft3* and *tsc1/tsc2*

**Fig. 5.**
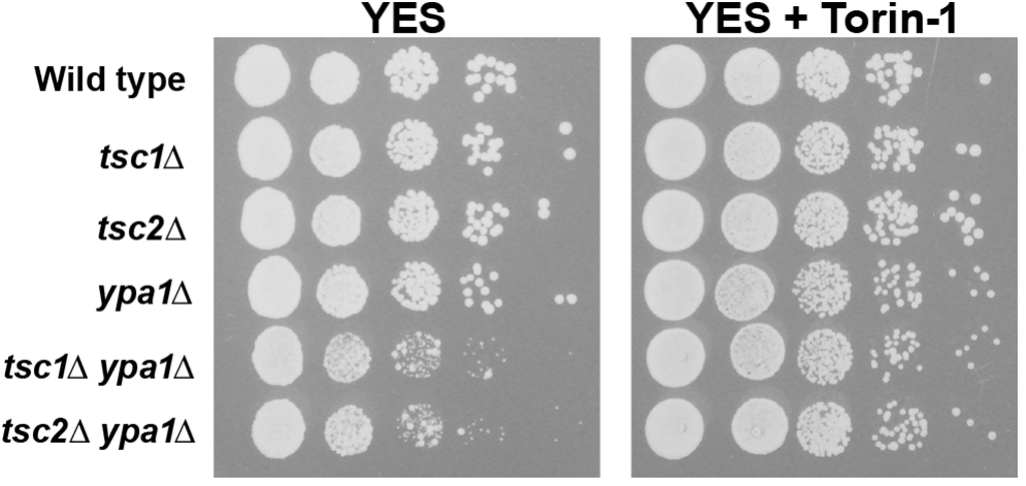
**Treatment with the mTOR inhibitor, torin-1, rescues the synthetic growth defect observed in *ypa1Δ tsc1/tsc2Δ* mutants.** Ten-fold serial dilutions of the indicated yeast cultures were plated on YES medium, or YES medium containing 750 nM torin-1, and incubated for 3 days at 30°C.

We were next interested in better understanding the mechanism(s) behind the negative interactions observed between *ypa1*/*fft3* and *tsc1*/*tsc2*. Since loss of *tsc1*/*tsc2* leads to abnormal hyper-activation of the mTOR pathway we reasoned that Ypa1p or Fft3p function might be specifically required when cells were ‘locked’ in a state of high mTOR activity. To test this hypothesis, we examined the growth of *ypa1Δ tsc1Δ*, *ypa1Δ tsc2Δ*, *fft3Δ tsc1Δ* and *fft3Δ tsc2Δ* double mutants on media containing the mTOR inhibitor torin-1. Remarkably, we found that torin-1 treatment rescued the synthetic growth defect observed in *ypa1Δ tsc1Δ* and *ypa1Δ tsc2Δ* double mutants (Fig. 5). Thus, the *ypa1*-specific negative interaction results from defects in the canonical functions of *tsc1* and *tsc2* (i.e. their role in regulating mTOR signaling). Since torin-1 treatment is epistatic to the effects of the *ypa1* deletion in *tsc1Δ/tsc2Δ* backgrounds, this result also strongly suggests that Ypa1p functions upstream of mTOR.

In contrast, torin-1 treatment had no effect on the negative interactions observed between *fft3 and tsc1/tsc2* (data not shown). This suggests that the *fft3*-specific negative interactions result from defects in a non-canonical function of the Tsc1p/Tsc2p complex (i.e. a function independent of its role in modulating mTOR signaling).

### *ypa1Δ* cells are resistant to the glycolytic inhibitor 2-deoxyglucose

The previous results suggested that the high metabolic activity conferred by hyperactive mTOR could not be tolerated in *ypa1Δ* mutant backgrounds. We thus examined whether or not *ypa1* itself affected metabolism. We began by growing *ypa1Δ* cells in the presence of the glycolytic inhibitor 2-deoxyglucose (2-DG). 2-DG is a glucose analog in which the hydroxyl group at C-2 is replaced with hydrogen. It is actively imported by hexose transporters and can be phosphorylated to produce 2-DG-6-phosphate. Unlike glucose-6-phosphate, this form cannot be further metabolized and accumulates within the cell, inhibiting glycolytic flux (possibly through product inhibition of hexokinase) and reducing cellular ATP pools (Vishwanatha and D'Souza, 2017).

**Fig. 6.**
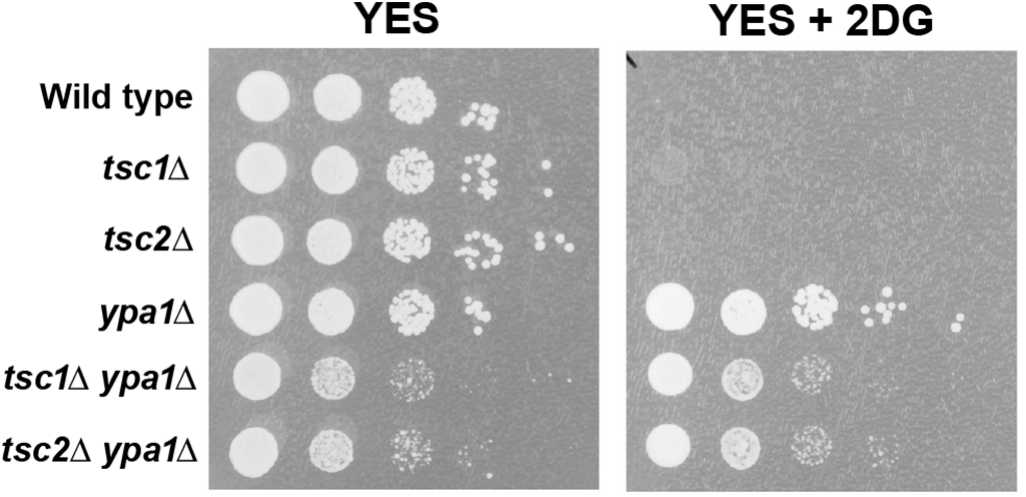
***ypa1Δ* cells are resistant to 2-DG.** Ten-fold serial dilutions of the indicated yeast cultures were plated on YES medium, or YES medium containing 1 mg/ml 2-DG, and incubated for 3 days at 30°C.

Remarkably, we found that *ypa1Δ* cells, as well as *ypa1Δ tsc1Δ* and *ypa1Δ tsc2Δ* double mutants, were highly resistant to 2-DG (Fig. 6). The observation that *ypa1Δ* mutants are resistant to the drug suggests that these cells have reduced glycolytic rates (i.e. they rely less on glycolysis to generate ATP). We suggest that this altered physiology, in conjunction with high mTOR levels brought about by the inactivation of Tsc1p/Tsc2p, leads to a conflicted and metabolically unbalanced state that profoundly inhibits growth (see Discussion).

## DISCUSSION

An understanding of any biological process (including disease processes) goes well beyond elucidating the function of genes in isolation (Hartman et al., 2001; Hartwell, 2004). In fact, research of the past decade has made it abundantly clear that complex genetic interactions (i.e. buffering relationships between genes) play a crucial role in modulating the genotype-phenotype relationship (Baryshnikova et al., 2013; Butland et al., 2008; Byrne et al., 2007; Costanzo et al., 2016; Housden et al., 2017). Fortunately, methodologies capable of characterizing genetic interactions at the genome-wide level have been developed to aid researchers in grappling with this complexity (Dixon et al., 2009b; Kuzmin et al., 2016). The present study sought to exploit such data to more fully elucidate the molecular pathology of *tsc1/tsc2* defects and to identify susceptibilities in cells lacking hamartin or tuberin function. This approach identified two *S. pombe* genes (*fft3* and *ypa1*), the individual loss of which confer growth defects in hamartin/tuberin-deficient cells.

### The molecular functions of *fft3*

The *fft3* gene encodes a Swi/Snf2 family ATP-dependent chromatin remodeler that is highly conserved in eukaryotes. Previous work has shown that Fft3p is essential in maintaining heterochromatic structure in centromeric and sub-telomeric regions and furthermore that it suppresses the turnover of histones at heterochromatic loci to facilitate the epigenetic transmission of heterochromatin (Steglich et al., 2015; Strålfors et al., 2011; Taneja et al., 2017; Taneja and Grewal, 2017). Deletion of *fft3* in *S. pombe* results in euchromatin formation, leading to incorrect histone modifications and the misregulation of gene expression (Strålfors et al., 2011).

Interestingly, Fft3p orthologs – Fun30 in *S. cerevisiae* and SMARCAD1 in humans – are known to aid in DNA end resection of double-stranded breaks (DSBs) via homologous recombination (Chen et al., 2012; Costelloe et al., 2012; Eapen et al., 2012). The inability to successfully repair DSBs compromises genome integrity and leads to prolonged G1/G2 checkpoint arrest, which often results in apoptosis (Massagué, 2004). Budding yeast Fun30 can increase the rate of 5′-to-3′ DNA resection and deactivate DNA damage checkpoint arrest (Eapen et al., 2012). This is done at the chromatin level, where Fun30 (as well as the human homolog SMARCAD1) can relax the tight histone-DNA interactions in nucleosomes adjacent to DSBs (Chen et al., 2012; Costelloe et al., 2012).

While Fft3p orthologs clearly play important roles in key cellular processes (transcription, DNA repair and DNA replication), the molecular mechanism by which the loss of *fft3* inhibits growth in *tsc1Δ* or *tsc2Δ* backgrounds in fission yeast remains unknown. The fact that the growth defect is not rescued by torin-1 treatment suggests that a non-canonical function of the Tsc1p/Tsc2p complex (i.e. a function independent of its role in modulating mTOR signaling) is involved. The synthetic negative interaction also implies one of two scenarios: (1) that *fft3* and *tsc1/tsc2* function in parallel pathways that impinge on a shared (and as yet unknown) function affecting growth; or (2) that the interaction reflects ‘unidirectional compensation’, a situation in which one pathway normally prevents a potentially harmful cellular event that can be corrected by another pathway (Boone et al., 2007).

### The molecular functions of *ypa1*

In contrast to *fft3*, much more is known with respect to *ypa1* and its potential molecular role in modulating TSC signaling. In fact, based on both the published literature and our own experimental results, we have developed a working model with respect to the role of the Ypa1p protein (and its orthologs) in regulating TSC and mTOR signaling. Several key observations have led to this model, which posits the existence of a novel signaling axis extending from the cellular sensing of glucose to downstream mTOR activation. These findings are discussed in detail below.

The first series of observations relate to the known role of budding yeast Ypa1p (also known as Rrd1p) in regulating protein phosphatase 2A (PP2A) activity in response to glucose. Briefly, work in the budding yeast has shown that the addition of glucose to glucose-starved cells triggers the post-translational activation of PP2A, that Ypa1p physically interacts with PP2A-like phosphatases, and that full glucose-mediated activation of PP2A is dependent on Ypa1p (Castermans et al., 2012; Fellner et al., 2003; Van Hoof et al., 2005). It is thus clearly established that Ypa1p is required for the glucose-induced activation of PP2A, at least in budding yeast.

The second series of observations relates to the role of PP2A in inhibiting the activation of AMP-activated protein kinase (AMPK). In *S. pombe*, just as in other more developmentally complex eukaryotes, AMPK plays a key role in the regulation of cellular energy homeostasis (through positively regulating energy-producing pathways and inhibiting energy-consuming processes in response to low ATP levels). *S. pombe* AMPK is composed of three subunits: an alpha subunit (Ssp2p), a beta subunit (Amk2p), and a gamma subunit (Cbs2p). It is clear that nutrient starvation (glucose or nitrogen) triggers Thr-189 phosphorylation of the Ssp2p subunit and the activation of AMPK (Valbuena and Moreno, 2010). Interestingly, it has also been shown that human PP2A negatively regulates AMPK by dephosphorylating Thr-172 (equivalent to Thr-189 in fission yeast). These researchers also showed (through co-immunoprecipitation and colocalization studies) that PP2A directly interacts with AMPK (Joseph et al., 2015). While not directly shown experimentally in fission yeast, these results raise the possibility that fission yeast PP2A might also regulate AMPK through modulating Thr-189 phosphorylation.

The last series of observations relates to the regulation of Tsc1p/Tsc2p and mTOR by AMPK. In human HEK293 cells, it is clear that AMPK phosphorylates Tsc2p (on Thr-1227 and Ser-1345) in response to energy starvation and that these phosphorylation events enhance the activity of Tsc2p with respect to inhibiting mTOR (Inoki et al., 2003). Similarly, in *S. pombe*, nutrient stress has been shown to activate the Ssp2p alpha subunit of AMPK and reduce mTOR activity in a manner that is dependent on both Tsc1p and Tsc2p (Davie et al., 2015). It thus appears that AMPK, Tsc1/Tsc2 and mTOR function as a conserved signaling module that is sensitive to cellular energy status.

**Fig. 7.**
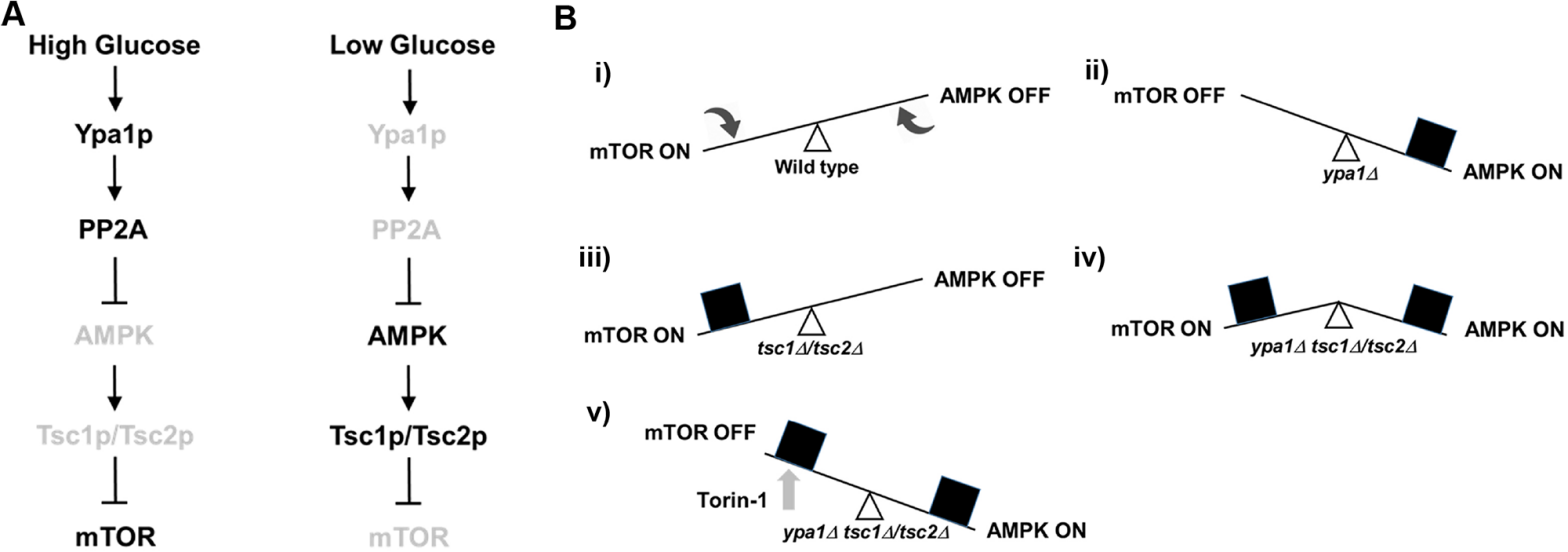
**Model describing the putative role of Ypa1p in mTOR signaling in fission yeast.** (A) Hypothesized signaling axis extending from the sensing of glucose levels via Ypa1p to the activation of mTOR. Components listed in black type are considered fully active. Components listed in grey type are considered minimally active. (B) Abstract model describing the effects of mutations in *ypa1* and *tsc1/tsc2*, both singly and in combination. (i) In the presence of glucose mTOR activity is high and AMPK activity is low. However, the system is free to shift in either direction as a function of glucose concentration. (ii) In *ypa1Δ* mutants, AMPK is locked in the ON position. The cell is maladapted, but the metabolic signals being output are consistent. (iii) In *tsc1Δ/tsc2Δ* mutants mTOR is locked in the ON position. The cell is maladapted, but again the metabolic signals being output are consistent. (iv) In *ypa1Δ tsc1Δ/tsc2Δ* mutants both mTOR and AMPK are locked in the ON position. The metabolic signals being output are contradictory, resulting in a synthetic growth defect. (v) Torin-1 treatment rescues the growth defect by turning mTOR OFF and restoring the balance.

Bringing these three lines of reasoning together, we propose the existence of a signaling axis leading from the sensing of glucose to the activation of mTOR (through the intermediaries, Ypa1p, PP2A, AMPK and Tsc1p/Tsc2p) (Fig. 7A). It is important to note that in this model, Tsc1p/Tsc2p acts as a critical go-between in relaying information between AMPK and mTOR. Since AMPK positively regulates Tsc1p/Tsc2p and Tsc1p/Tsc2p negatively regulates mTOR, this regulatory design ensures that AMPK activity and mTOR activity are inverted with respect to each other (i.e. high AMPK leads to low mTOR, and low AMPK leads to high mTOR). We suggest that the role of Tsc1p/Tsc2p in balancing these two activities is crucial in ensuring that the cell maintains the proper balance between the nutrient-induced and starvation-induced activities of AMPK and mTOR. This is to say, in the presence of Tsc1p/Tsc2p, high catabolic rates (high AMPK) correlate with a starvation response (low mTOR). In contrast, high anabolic rates (low AMPK) correlate with the promotion of cell growth/proliferation (high mTOR). Finally, moderate catabolic rates (intermediate AMPK) correlate with a moderate starvation response (intermediate mTOR) (Fig. 7B).

Importantly, while mutants lacking either Ypa1p or Tsc1p/Tsc2p activity would indeed be maladapted, such cells would still maintain a ‘balance’ between AMPK and mTOR activity. For instance, in *ypa1Δ* mutants grown in nutrient rich conditions, AMPK would be abnormally locked in the ON position and mTOR in the OFF position. Likewise, in *tsc1Δ/tsc2Δ* mutants grown in nutrient poor conditions, AMPK would be abnormally locked in the OFF position and mTOR in the ON position. While maladapted, both cell types are consistent with respect to the metabolic signals being output to the cell (Fig. 7B).

Finally, consider the scenario in which cells lacks both Ypa1p and Tsc1p/Tsc2p activity (resulting in both AMPK and mTOR being locked in the ON position). In this case, the signals emanating from AMPK and mTOR are in direct contradiction; with AMPK promoting catabolic activity at the same time mTOR is promoting cell growth and proliferation (Fig. 7B). We hypothesize that the ‘conflicted and metabolically unbalanced’ cellular state characterized by the loss of both Ypa1p and Tsc1p/Tsc2p activity is the root cause of the synthetic growth defect observed in cells bearing loss-of-function mutations in both *ypa1* and *tsc1/tsc2*. Importantly, it should be noted that the model predicts that the observed synthetic growth defect would be rescued by mTOR inhibition (by placing the cell back in a ‘balanced’ state) (Fig. 7B). As previously discussed, this is indeed the case as torin-1 treatment was seen to dramatically improve the growth of double mutant cells (Fig. 5). Lastly, and perhaps most intriguingly, these results also imply that cells devoid of hamartin/tuberin function would be especially vulnerable to treatments that directly promote AMPK activity.

Given this last point, it is interesting to speculate as to the potential conservation of the observed *fft3*- or *ypa1*-specific negative interactions in more developmentally complex eukaryotes. This is to say, it would be interesting to determine whether RNAi knockdown of either *SMARCAD1* (human *fft3* ortholog) or *PPP2R4* (human *ypa1* ortholog) in *TSC1*- or *TSC2*-deficient human cell lines would also result in reduced growth rates. If indeed conserved, then this raises the question of whether it might be possible to inhibit the growth of hamartin/tuberin deficient tumor cells by recapitulating the phenotypic effects of *SMARCAD1* or *PPP2R4* loss of function. In any event, it is hoped that a detailed understanding of *ypa1* or *fft3* function in fission yeast might reveal foundational knowledge that could be leveraged to reveal a previously unrecognized Achilles' heel exhibited by cells lacking hamartin/tuberin activity.

## MATERIALS AND METHODS

### Strains, media and growth conditions

*S. pombe* cells were cultured in YES media or EMM media supplemented as needed with adenine, histidine, leucine and/or uracil (Forsburg and Rhind, 2006). Liquid cultures were grown with shaking (200 rpm) at 30°C. All fission yeast strains used in this study were either derived from the Karagiannis laboratory collection, constructed during the course of this work (described below), or purchased from Bioneer Corporation as part of version 4 of the haploid gene deletion mutant library (Kim et al., 2010). All genetic crosses and general yeast techniques were performed using standard methods (Forsburg and Rhind, 2006).

### Bioinformatics

Ortholog prediction and alignment was performed using the DRSC Integrative Ortholog Prediction Tool Version 5.5 (http://www.flyrnai.org/cgi-bin/DRSC_orthologs.pl) using human TSC1 or human TSC2 as the input species and *S. pombe* as the output species. Scores less than one were excluded. Details of the tool can be found in Hu et al. (2011).

Genetic interaction network analysis was performed using the BioGRID webtool (http://thebiogrid.org/) using *S. pombe tsc1* or *tsc2* as input. Data were filtered by excluding both physical and chemical interactions, and by excluding genetic interactions supported by only one experimental result. Network diagrams were created using the embedded BioGRID webtool powered by CytoscapeJS using the Arbor layout. Details of the tool can be found in Chatr-Aryamontri et al. (2017).

### Colony size assays

A Axioskop 40 micromanipulator (Zeiss, Oberkochen, Germany) was used to array single cells representing each genotype (in quadruplicate) upon YES agar plates. Plates were incubated at 30°C for 5 days. Digital images were taken at 24 h intervals. Colony size was determined by measuring the area of each colony from the images using the ‘measure’ tool of ImageJ (http://imagej.nih.gov/ij/). Statistically significant differences in colony size among the genotypes were determined using the non-parametric Wilcoxon matched pairs signed rank test (two-tailed). Three biological replicates were performed for each tested interaction.

### ε-value calculations

Using colony size as a proxy for fitness, ε-values were calculated by subtracting the expected double mutant fitness from the observed double mutant fitness, ε=*W_ab_*−*E(W_ab_)*. The expected fitness was calculated using one of four commonly used neutrality models. In the Product model, *E(W_ab_)*=*W_a_ W_b_*. In the Min model, *E(W_ab_)*=*min*(*W_a_*, *W_b_)*. In the Log model, *E(W_ab_)*=log_2_[(2*^Wa^*−1) (2*^Wb^*−1)+1]. In the Additive model, *E(W_ab_)*=*W_a_*+*W_b_*−1.

### Spot assays

Strains of the indicated genotype were grown overnight at 30°C in liquid YES medium to an optical density of 0.5. Five microliters of undiluted culture, as well as four 10-fold serial dilutions (made in liquid YES), were then spotted onto YES-agar plates, or YES-agar plates containing 60 µg/ml canavanine (C-1625, Sigma-Aldrich), 750 nM torin-1 (A11587, AdooQ Bioscience, Irvine, USA), or 1 mg/ml 2-deoxyglucose (D-6134, Sigma-Aldrich). Growth was assayed visually after the plates had been incubated for 3 days at 30°C.

### Construction of *tsc1* and *tsc2* gene deletion strains

Gene deletion cassettes for both the *tsc1* and *tsc2* genes were created by polymerase chain reaction (PCR) amplifying the *ura4*^+^ selectable marker (together with the *loxP* and *loxM3* recombination sites) from the pAW1 vector using primers tsc11 (5′-TTA TCA ATG CTG CCA AGA CTT GCT ATC AGT ATA ATG TCG CAT AGT TGT ATA TCA ACG TTG ACT TTG CCA ACT TTG TAC GAC GGA TCC CCG GGT TAA TTA A-3′) and tsc12 (5′-AAT TAT TTT ATA TGG AAT GAG CAA GTA TGT TTT ATC ATA ATT GAC CAG TTC ATT TCA AGG ACC TTC AAA AAT ATA CCT ACG AAT TCG AGC TCG TTT AAA C-3′), or tsc13 (5′-TTA AGA GTT CAG ATT TGC TTT ATG TGG TTA TTC TGC TGA AGG TCC TAA TTT ATT GAC GTT GAA AAA TAA AGG CCA CAT AGC GGA TCC CCG GGT TAA TTA A-3′) and tsc14 (5′-ATA AAA ATT AAT TAA TGA TGG CAA GGC ACA ATC GTA ATC AAT CTT TTA ATT TAG GAC TTT TTA TAT GCC CTT ATG GCG AAT TCG AGC TCG TTT AAA C-3′), respectively. Strain ED666 (*ura4-D18 leu1-32 ade6-210 h^+^*) was then transformed with either the *tsc1*-specific or *tsc2*-specific cassette. Ura^+^ transformants were isolated and the respective gene deletions confirmed by colony PCR using primers tsc15 (5′-ATG TGG CAG ACT ACG CTA TCC T-3′) and tsc17 (5′-ATG CTT CCC CTA ATT CAT AGC A-3′) for the *tsc1* deletion, or tsc16 (5′-AGC AAC CTA CGA GAG GAA GAT G-3′) and tsc18 (5′-GCG CAT AAC CCT TTC TAC ATT C-3′) for the *tsc2* deletion.

### Construction of fission yeast strains bearing site-directed mutations in *fft3* or *ypa1*

C-terminal fragments of the *fft3* and *ypa1* genes bearing the desired mutations were synthesized by GenScript and cloned into the *S. pombe* integration vector, pJK210, using the *EcoRI* and *SmaI* sites, or the *XhoI* and *SmaI* sites, respectively. The pJK210-fft3-K418R and pJK210-fft3-Δ791-872 constructs were linearized within the *fft3* sequence (upstream of the relevant mutations) using *BglII* and then transformed into a *ura4-D18* strain using the lithium acetate protocol (Forsburg and Rhind, 2006). The pJK210-ypa1-D212G construct was similarly linearized with *BstBI* before being transformed into a *ura4-D18* strain using the lithium acetate method. Ura4^+^ transformants were then assessed for integration at the *fft3* or *ypa1* loci through colony PCR. Proper integration at the *fft3* locus was assessed using primers AR2F (5′-TCC CTC ATT TAC TTC CTC TGC TAA-3′) and AR2R (5′-TCC TAT GTT GTG TGG AAT TGT GAG-3′). Proper integration at the *ypa1* locus was assessed using primers AR1F (5′-TAC AGC GTC TCA ATA TTG CAT CTG-3′) and AR1R (5′-TCC TAT GTT GTG TGG AAT TGT GAG-3′).

### Recombinase-mediated cassette exchange of human *TSC1* and *TSC2* sequences at the *S. pombe tsc1* or *tsc2* loci

The full-length human *TSC1* or *TSC2* sequences were synthesized by Genscript and cloned into the *XhoI* and *SpeI* sites of the pAW8X vector (Watson et al., 2008). The *tsc1::ura4^+^/tsc2::ura4^+^* base strains were then transformed with the pAW8X-TSC1/pAW8X-TSC2 vectors using the lithium acetate protocol (Forsburg and Rhind, 2006). As controls, the base strains were also transformed with the pAW8X-tsc1 and pAW8X-tsc2 vectors containing the fission yeast *tsc1* and *tsc2* genes. Leu^+^ transformants were then grown in YES media to allow the plasmid to be lost. Leu^−^ cells in which the *ura4^+^* gene was exchanged with *TSC1*/*TSC2*/*tsc1*/*tsc2* were selected by growth on media containing 5-fluoroorotic acid (a drug counter-selectable to Ura^+^ cells). This created strains in which human *TSC1* or *TSC2*, or fission yeast *tsc1* or *tsc2*, were expressed from the endogenous fission yeast *tsc1*/*tsc2* promoters at the native *tsc1*/*tsc2* loci.

### Construction of fission yeast strains bearing site-directed mutations in *tsc1* or *tsc2*

The full-length *S. pombe tsc1* or *tsc2* genes (flanked by *XhoI* and *SpeI* sites) were synthesized by Genscript and cloned into the *EcoRV* site of the pUC57-vector. A Q5 Site-Directed Mutagenesis Kit (New England Biolabs, Ipswich, USA) was then used according to the manufacturer's protocol to generate the pUC57-tsc1-L346H, pUC57-tsc1-G432R, pUC57-tsc2-G296E, pUC57-tsc2-N1191*K*, pUC57-tsc2-N1199S and pUC57-tsc2-R1296P vectors. Mutagenic primers were designed using the NEBaseChanger webtool (http://nebasechanger.neb.com). DNA sequence analysis confirmed the incorporation of the desired changes. The respective *tsc1* or *tsc2* sequences were then digested from the pUC57 parent vector and cloned into the *XhoI* and *SpeI* sites of the pAW8X exchange vector. The *tsc1::ura4^+^/tsc2::ura4^+^* base strains were then individually transformed with the pAW8X-tsc1-G432R, pAW8X-tsc2-G296E, pAW8X-tsc2-N1191*K*, pAW8X-tsc2-N1199S and pAW8X-tsc2-R1296P vectors using the lithium acetate protocol (Forsburg and Rhind, 2006). Leu^+^ transformants were then grown in YES medium to allow the plasmid to be lost. Leu^−^ cells in which the *ura4^+^* gene was exchanged with the *tsc1*/*tsc2* mutants were selected by growth on medium containing 5-fluoroorotic acid (a drug counter-selectable to Ura^+^ cells). This created strains in which the respective *tsc1* or *tsc2* mutants were expressed from the endogenous fission yeast *tsc1*/*tsc2* promoters at the native *tsc1*/*tsc2* loci.

## Supplementary Material

Supplementary information

## References

[BIO031302C1] Aspuria P.-J. and Tamanoi F. (2008). The Tsc/Rheb signaling pathway controls basic amino acid uptake via the Cat1 permease in fission yeast. *Mol. Genet. Genomics* 279, 441-450. 10.1007/s00438-008-0320-y 18219492 PMC2670428

[BIO031302C2] Atias N., Kupiec M. and Sharan R. (2016). Systematic identification and correction of annotation errors in the genetic interaction map of *Saccharomyces cerevisiae*. *Nucleic Acids Res.* 44, e50 10.1093/nar/gkv1284 26602688 PMC4797274

[BIO031302C3] Baryshnikova A. and Andrews B. (2012). Neighboring-gene effect: a genetic uncertainty principle. *Nat. Methods* 9, 341-343. 10.1038/nmeth.1936 22453910

[BIO031302C4] Baryshnikova A., Costanzo M., Myers C. L., Andrews B. and Boone C. (2013). Genetic interaction networks: toward an understanding of heritability. *Annu. Rev. Genomics Hum. Genet.* 14, 111-133. 10.1146/annurev-genom-082509-141730 23808365

[BIO031302C5] Ben-Shitrit T., Yosef N., Shemesh K., Sharan R., Ruppin E. and Kupiec M. (2012). Systematic identification of gene annotation errors in the widely used yeast mutation collections. *Nat. Methods* 9, 373-378. 10.1038/nmeth.1890 22306811

[BIO031302C6] Boone C., Bussey H. and Andrews B. J. (2007). Exploring genetic interactions and networks with yeast. *Nat. Rev. Genet.* 8, 437-449. 10.1038/nrg2085 17510664

[BIO031302C7] Butland G., Babu M., Díaz-Mejía J. J., Bohdana F., Phanse S., Gold B., Yang W., Li J., Gagarinova A. G., Pogoutse O. et al. (2008). eSGA: *E. coli* synthetic genetic array analysis. *Nat. Methods* 5, 789-795. 10.1038/nmeth.1239 18677321

[BIO031302C8] Byrne A. B., Weirauch M. T., Wong V., Koeva M., Dixon S. J., Stuart J. M. and Roy P. J. (2007). A global analysis of genetic interactions in *Caenorhabditis elegans*. *J. Biol.* 6, 8 10.1186/jbiol58 17897480 PMC2373897

[BIO031302C9] Castermans D., Somers I., Kriel J., Louwet W., Wera S., Versele M., Janssens V. and Thevelein J. M. (2012). Glucose-induced posttranslational activation of protein phosphatases PP2A and PP1 in yeast. *Cell Res.* 22, 1058-1077. 10.1038/cr.2012.20 22290422 PMC3367521

[BIO031302C10] Chatr-Aryamontri A., Oughtred R., Boucher L., Rust J., Chang C., Kolas N. K., O'Donnell L., Oster S., Theesfeld C., Sellam A. et al. (2017). The BioGRID interaction database: 2017 update. *Nucleic Acids Res.* 45, D369-D379. 10.1093/nar/gkw1102 27980099 PMC5210573

[BIO031302C11] Chen X., Cui D., Papusha A., Zhang X., Chu C.-D., Tang J., Chen K., Pan X. and Ira G. (2012). The Fun30 nucleosome remodeller promotes resection of DNA double-strand break ends. *Nature* 489, 576-580. 10.1038/nature11355 22960743 PMC3640768

[BIO031302C12] Costanzo M., VanderSluis B., Koch E. N., Baryshnikova A., Pons C., Tan G., Wang W., Usaj M., Hanchard J., Lee S. D. et al. (2016). A global genetic interaction network maps a wiring diagram of cellular function. *Science* 353, aaf1420 10.1126/science.aaf1420 27708008 PMC5661885

[BIO031302C13] Costelloe T., Louge R., Tomimatsu N., Mukherjee B., Martini E., Khadaroo B., Dubois K., Wiegant W. W., Thierry A., Burma S. et al. (2012). The yeast Fun30 and human SMARCAD1 chromatin remodellers promote DNA end resection. *Nature* 489, 581-584. 10.1038/nature11353 22960744 PMC3493121

[BIO031302C14] Davie E., Forte G. M. A. and Petersen J. (2015). Nitrogen regulates AMPK to control TORC1 signaling. *Curr. Biol.* 25, 445-454. 10.1016/j.cub.2014.12.034 25639242 PMC4331286

[BIO031302C15] Dixon S. J., Fedyshyn Y., Koh J. L. Y., Prasad T. S. K., Chahwan C., Chua G., Toufighi K., Baryshnikova A., Hayles J., Hoe K.-L. et al. (2008). Significant conservation of synthetic lethal genetic interaction networks between distantly related eukaryotes. *Proc. Natl. Acad. Sci. USA* 105, 16653-16658. 10.1073/pnas.0806261105 18931302 PMC2575475

[BIO031302C16] Dixon S. J., Andrews B. J. and Boone C. (2009a). Exploring the conservation of synthetic lethal genetic interaction networks. *Commun. Integr. Biol.* 2, 78-81. 10.4161/cib.7501 19704894 PMC2686349

[BIO031302C17] Dixon S. J., Costanzo M., Baryshnikova A., Andrews B. and Boone C. (2009b). Systematic mapping of genetic interaction networks. *Annu. Rev. Genet.* 43, 601-625. 10.1146/annurev.genet.39.073003.114751 19712041

[BIO031302C18] Eapen V. V., Sugawara N., Tsabar M., Wu W.-H. and Haber J. E. (2012). The *Saccharomyces cerevisiae* chromatin remodeler Fun30 regulates DNA end resection and checkpoint deactivation. *Mol. Cell. Biol.* 32, 4727-4740. 10.1128/MCB.00566-12 23007155 PMC3486187

[BIO031302C19] Fellner T., Lackner D. H., Hombauer H., Piribauer P., Mudrak I., Zaragoza K., Juno C. and Ogris E. (2003). A novel and essential mechanism determining specificity and activity of protein phosphatase 2A (PP2A) in vivo. *Genes Dev.* 17, 2138-2150. 10.1101/gad.259903 12952889 PMC196455

[BIO031302C20] Forsburg S. L. and Rhind N. (2006). Basic methods for fission yeast. *Yeast* 23, 173-183. 10.1002/yea.1347 16498704 PMC5074380

[BIO031302C21] Goyal A. and Simanis V. (2012). Characterization of *ypa1* and *ypa2*, the *Schizosaccharomyces pombe* orthologs of the peptidyl proyl isomerases that activate PP2A, reveals a role for Ypa2p in the regulation of cytokinesis. *Genetics* 190, 1235-1250. 10.1534/genetics.111.138040 22267499 PMC3316640

[BIO031302C22] Guo F., Stanevich V., Wlodarchak N., Sengupta R., Jiang L., Satyshur K. A. and Xing Y. (2014). Structural basis of PP2A activation by PTPA, an ATP-dependent activation chaperone. *Cell Res.* 24, 190-203. 10.1038/cr.2013.138 24100351 PMC3915903

[BIO031302C23] Hartman J. L. IV, Garvik B. and Hartwell L. (2001). Principles for the buffering of genetic variation. *Science* 291, 1001-1004. 10.1126/science.291.5506.1001 11232561

[BIO031302C24] Hartwell L. (2004). GENETICS: robust interactions. *Science* 303, 774-775. 10.1126/science.1094731 14764857

[BIO031302C25] Henske E. P., Jóźwiak S., Kingswood J. C., Sampson J. R. and Thiele E. A. (2016). Tuberous sclerosis complex. *Nat. Rev. Dis. Primer.* 2, 16035 10.1038/nrdp.2016.35 27226234

[BIO031302C26] Hoffman C. S., Wood V. and Fantes P. A. (2015). An ancient yeast for young geneticists: a primer on the *Schizosaccharomyces pombe* model system. *Genetics* 201, 403-423. 10.1534/genetics.115.181503 26447128 PMC4596657

[BIO031302C27] Housden B. E., Valvezan A. J., Kelley C., Sopko R., Hu Y., Roesel C., Lin S., Buckner M., Tao R., Yilmazel B. et al. (2015). Identification of potential drug targets for tuberous sclerosis complex by synthetic screens combining CRISPR-based knockouts with RNAi. *Sci. Signal.* 8, rs9 10.1126/scisignal.aab3729 26350902 PMC4642709

[BIO031302C28] Housden B. E., Nicholson H. E. and Perrimon N. (2017). Synthetic lethality screens using RNAi in combination with CRISPR-based knockout in Drosophila cells. *Bio-Protoc.* 7, e2119 10.21769/BioProtoc.2119 28523286 PMC5432019

[BIO031302C29] Hu Y., Flockhart I., Vinayagam A., Bergwitz C., Berger B., Perrimon N. and Mohr S. E. (2011). An integrative approach to ortholog prediction for disease-focused and other functional studies. *BMC Bioinformatics* 12, 357 10.1186/1471-2105-12-357 21880147 PMC3179972

[BIO031302C30] Inoki K., Li Y., Xu T. and Guan K.-L. (2003). Rheb GTPase is a direct target of TSC2 GAP activity and regulates mTOR signaling. *Genes Dev.* 17, 1829-1834. 10.1101/gad.1110003 12869586 PMC196227

[BIO031302C31] Jordens J., Janssens V., Longin S., Stevens I., Martens E., Bultynck G., Engelborghs Y., Lescrinier E., Waelkens E., Goris J. et al. (2006). The protein phosphatase 2A phosphatase activator is a novel peptidyl-prolyl cis/trans-isomerase. *J. Biol. Chem.* 281, 6349-6357. 10.1074/jbc.M507760200 16380387

[BIO031302C32] Joseph B. K., Liu H.-Y., Francisco J., Pandya D., Donigan M., Gallo-Ebert C., Giordano C., Bata A. and Nickels J. T. (2015). Inhibition of AMP kinase by the protein phosphatase 2A heterotrimer, PP2A^Ppp2r2d^. *J. Biol. Chem.* 290, 10588-10598. 10.1074/jbc.M114.626259 25694423 PMC4409226

[BIO031302C33] Kim D.-U., Hayles J., Kim D., Wood V., Park H.-O., Won M., Yoo H.-S., Duhig T., Nam M., Palmer G. et al. (2010). Analysis of a genome-wide set of gene deletions in the fission yeast *Schizosaccharomyces pombe*. *Nat. Biotechnol.* 28, 617-623. 10.1038/nbt.1628 20473289 PMC3962850

[BIO031302C34] Kuzmin E., Costanzo M., Andrews B. and Boone C. (2016). Synthetic genetic array analysis. *Cold Spring Harb. Protoc.* 2016, pdb.prot088807 10.1101/pdb.prot088807 27037072

[BIO031302C35] Lee J., Choi E. S., Seo H. D., Kang K., Gilmore J. M., Florens L., Washburn M. P., Choe J., Workman J. L. and Lee D. (2017). Chromatin remodeller Fun30Fft3 induces nucleosome disassembly to facilitate RNA polymerase II elongation. *Nat. Commun.* 8, 14527 10.1038/ncomms14527 28218250 PMC5321744

[BIO031302C36] Leulliot N., Vicentini G., Jordens J., Quevillon-Cheruel S., Schiltz M., Barford D., van Tilbeurgh H. and Goris J. (2006). Crystal structure of the PP2A phosphatase activator: implications for its PP2A-specific PPIase activity. *Mol. Cell* 23, 413-424. 10.1016/j.molcel.2006.07.008 16885030

[BIO031302C37] Li J., Kim S. G. and Blenis J. (2014). Rapamycin: one drug, many effects. *Cell Metab.* 19, 373-379. 10.1016/j.cmet.2014.01.001 24508508 PMC3972801

[BIO031302C38] Mani R., St Onge R. P., Hartman J. L., Giaever G. and Roth F. P. (2008). Defining genetic interaction. *Proc. Natl. Acad. Sci. USA* 105, 3461-3466. 10.1073/pnas.0712255105 18305163 PMC2265146

[BIO031302C39] Massagué J. (2004). G1 cell-cycle control and cancer. *Nature* 432, 298-306. 10.1038/nature03094 15549091

[BIO031302C40] Matsumoto S., Bandyopadhyay A., Kwiatkowski D. J., Maitra U. and Matsumoto T. (2002). Role of the Tsc1-Tsc2 complex in signaling and transport across the cell membrane in the fission yeast *Schizosaccharomyces pombe*. *Genetics* 161, 1053-1063. 12136010 10.1093/genetics/161.3.1053 PMC1462175

[BIO031302C41] Medvetz D., Priolo C. and Henske E. P. (2015). Therapeutic targeting of cellular metabolism in cells with hyperactive mTORC1: a paradigm shift. *Mol. Cancer Res.* 13, 3-8. 10.1158/1541-7786.MCR-14-0343 25298408 PMC4312527

[BIO031302C42] Nakase Y., Nakase M., Kashiwazaki J., Murai T., Otsubo Y., Mabuchi I., Yamamoto M., Takegawa K. and Matsumoto T. (2013). The fission yeast β-arrestin-like protein Any1 is involved in TSC-Rheb signaling and the regulation of amino acid transporters. *J. Cell Sci.* 126, 3972-3981. 10.1242/jcs.128355 23813957

[BIO031302C43] Neuman N. A. and Henske E. P. (2011). Non-canonical functions of the tuberous sclerosis complex-Rheb signalling axis. *EMBO Mol. Med.* 3, 189-200. 10.1002/emmm.201100131 21412983 PMC3377068

[BIO031302C44] Randle S. C. (2017). Tuberous sclerosis complex: a review. *Pediatr. Ann.* 46, e166-e171. 10.3928/19382359-20170320-01 28414398

[BIO031302C45] Rosset C., Netto C. B. O. and Ashton-Prolla P. (2017). *TSC1* and *TSC2* gene mutations and their implications for treatment in Tuberous Sclerosis Complex: a review. *Genet. Mol. Biol.* 40, 69-79. 10.1590/1678-4685-gmb-2015-0321 28222202 PMC5409767

[BIO031302C46] Ryan C. J., Roguev A., Patrick K., Xu J., Jahari H., Tong Z., Beltrao P., Shales M., Qu H., Collins S. R. et al. (2012). Hierarchical modularity and the evolution of genetic interactomes across species. *Mol. Cell* 46, 691-704. 10.1016/j.molcel.2012.05.028 22681890 PMC3380636

[BIO031302C47] Saxton R. A. and Sabatini D. M. (2017). mTOR signaling in growth, metabolism, and disease. *Cell* 169, 361-371. 10.1016/j.cell.2017.03.035 28388417

[BIO031302C48] Shen J. P., Zhao D., Sasik R., Luebeck J., Birmingham A., Bojorquez-Gomez A., Licon K., Klepper K., Pekin D., Beckett A. N. et al. (2017). Combinatorial CRISPR-Cas9 screens for de novo mapping of genetic interactions. *Nat. Methods* 14, 573-576. 10.1038/nmeth.4225 28319113 PMC5449203

[BIO031302C49] Stark C., Breitkreutz B.-J., Reguly T., Boucher L., Breitkreutz A. and Tyers M. (2006). BioGRID: a general repository for interaction datasets. *Nucleic Acids Res.* 34, D535-D539. 10.1093/nar/gkj109 16381927 PMC1347471

[BIO031302C50] Steglich B., Strålfors A., Khorosjutina O., Persson J., Smialowska A., Javerzat J.-P. and Ekwall K. (2015). The Fun30 chromatin remodeler Fft3 controls nuclear organization and chromatin structure of insulators and subtelomeres in fission yeast. *PLoS Genet.* 11, e1005101 10.1371/journal.pgen.1005101 25798942 PMC4370569

[BIO031302C51] Strålfors A., Walfridsson J., Bhuiyan H. and Ekwall K. (2011). The FUN30 chromatin remodeler, Fft3, protects centromeric and subtelomeric domains from euchromatin formation. *PLoS Genet.* 7, e1001334 10.1371/journal.pgen.1001334 21437270 PMC3060074

[BIO031302C52] Switon K., Kotulska K., Janusz-Kaminska A., Zmorzynska J. and Jaworski J. (2016). Tuberous sclerosis complex: from molecular biology to novel therapeutic approaches. *IUBMB Life* 68, 955-962. 10.1002/iub.1579 27797139

[BIO031302C53] Taneja N. and Grewal S. I. S. (2017). Shushing histone turnover: it's FUN protecting epigenome-genome. *Cell Cycle* 16, 1731-1732. 10.1080/15384101.2017.1360651 28805495 PMC5628654

[BIO031302C54] Taneja N., Zofall M., Balachandran V., Thillainadesan G., Sugiyama T., Wheeler D., Zhou M. and Grewal S. I. S. (2017). SNF2 family protein Fft3 suppresses nucleosome turnover to promote epigenetic inheritance and proper replication. *Mol. Cell* 66, 50-62.e6. 10.1016/j.molcel.2017.02.006 28318821 PMC5407362

[BIO031302C55] Tosti E., Katakowski J. A., Schaetzlein S., Kim H.-S., Ryan C. J., Shales M., Roguev A., Krogan N. J., Palliser D., Keogh M.-C. et al. (2014). Evolutionarily conserved genetic interactions with budding and fission yeast MutS identify orthologous relationships in mismatch repair-deficient cancer cells. *Genome Med.* 6, 68 10.1186/s13073-014-0068-4 25302077 PMC4189729

[BIO031302C56] Valbuena N. and Moreno S. (2010). TOR and PKA pathways synergize at the level of the Ste11 transcription factor to prevent mating and meiosis in fission yeast. *PloS ONE* 5, e11514 10.1371/journal.pone.0011514 20634885 PMC2901329

[BIO031302C57] Van Hoof C., Martens E., Longin S., Jordens J., Stevens I., Janssens V. and Goris J. (2005). Specific interactions of PP2A and PP2A-like phosphatases with the yeast PTPA homologues, Ypa1 and Ypa2. *Biochem. J.* 386, 93-102. 10.1042/BJ20040887 15447631 PMC1134770

[BIO031302C58] van Slegtenhorst M., Carr E., Stoyanova R., Kruger W. D. and Henske E. P. (2004). *tsc1*^+^ and *tsc2*^+^ regulate arginine uptake and metabolism in *Schizosaccharomyces pombe*. *J. Biol. Chem.* 279, 12706-12713. 10.1074/jbc.M313874200 14718525

[BIO031302C59] van Slegtenhorst M., Mustafa A. and Henske E. P. (2005). Pas1, a G1 cyclin, regulates amino acid uptake and rescues a delay in G1 arrest in Tsc1 and Tsc2 mutants in S*chizosaccharomyces pombe*. *Hum. Mol. Genet.* 14, 2851-2858. 10.1093/hmg/ddi317 16115814

[BIO031302C60] Vishwanatha A. and D'Souza C. J. M. (2017). Multifaceted effects of antimetabolite and anticancer drug, 2-deoxyglucose on eukaryotic cancer models budding and fission yeast. *IUBMB Life* 69, 137-147. 10.1002/iub.1599 28093891

[BIO031302C61] Watson A. T., Garcia V., Bone N., Carr A. M. and Armstrong J. (2008). Gene tagging and gene replacement using recombinase-mediated cassette exchange in *Schizosaccharomyces pombe*. *Gene* 407, 63-74. 10.1016/j.gene.2007.09.024 18054176

[BIO031302C62] Weisman R., Roitburg I., Nahari T. and Kupiec M. (2005). Regulation of leucine uptake by *tor1*^+^ in *Schizosaccharomyces pombe* is sensitive to rapamycin. *Genetics* 169, 539-550. 10.1534/genetics.104.034983 15466417 PMC1449110

[BIO031302C63] Weisman R., Roitburg I., Schonbrun M., Harari R. and Kupiec M. (2007). Opposite effects of *tor1* and *tor2* on nitrogen starvation responses in fission yeast. *Genetics* 175, 1153-1162. 10.1534/genetics.106.064170 17179073 PMC1840069

