# Supplementary material for: Using genetic buffering relationships identified in fission yeast to reveal susceptibilities in cells lacking hamartin or tuberin function
Source: Biol Open. 2017 Dec 20;7(1):bio031302. doi: 10.1242/bio.031302 (PMC5827267; doi:10.1242/bio.031302)
Supplement: Supplementary information [file biolopen-7-031302-s1.pdf]

SUPPLEMENTARY FIGURES

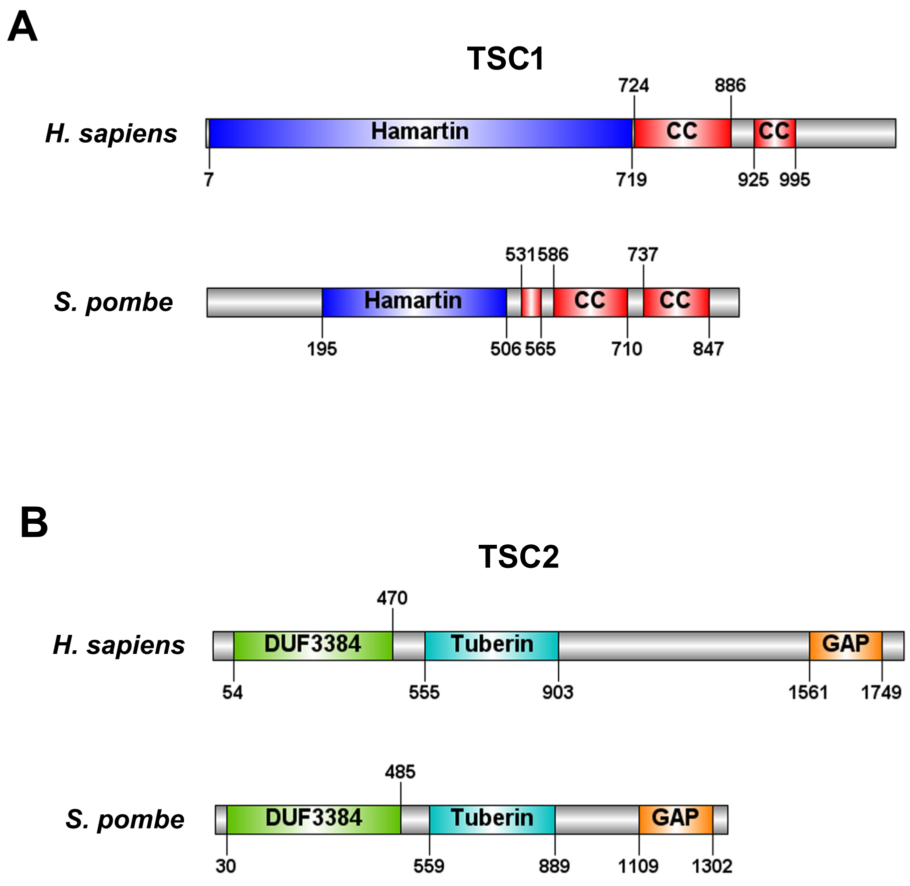

Figure S1 - Domain structure of human *TSC1* and *TSC2* in comparison to their respective fission yeast orthologs. CC, Coiled Coil; GAP, GTPase-activating protein; DUF, Domain of unknown function.

Biology Open • Supplementary information

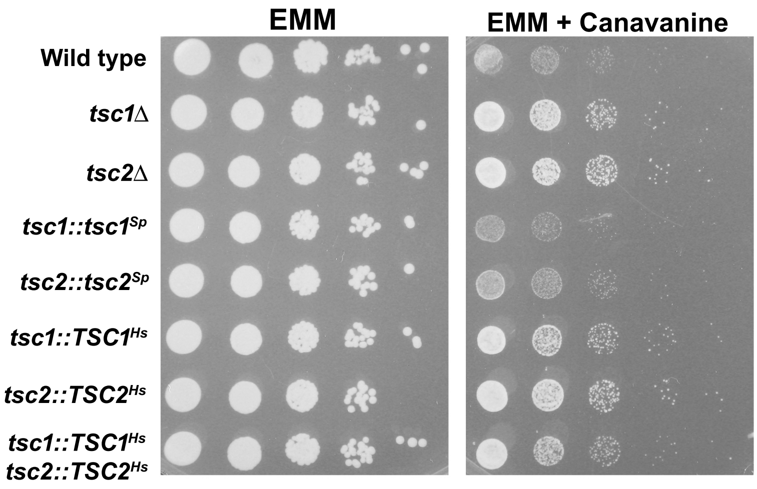

Figure S2 – The human *TSC1* or *TSC2* genes are unable to complement the loss of fission yeast *tsc1* or *tsc2*, respectively. Ten-fold serial dilutions of the indicated yeast cultures were plated on YES media, or YES media containing 60 µg/ml canavanine, and incubated for three days at 30°C.

A

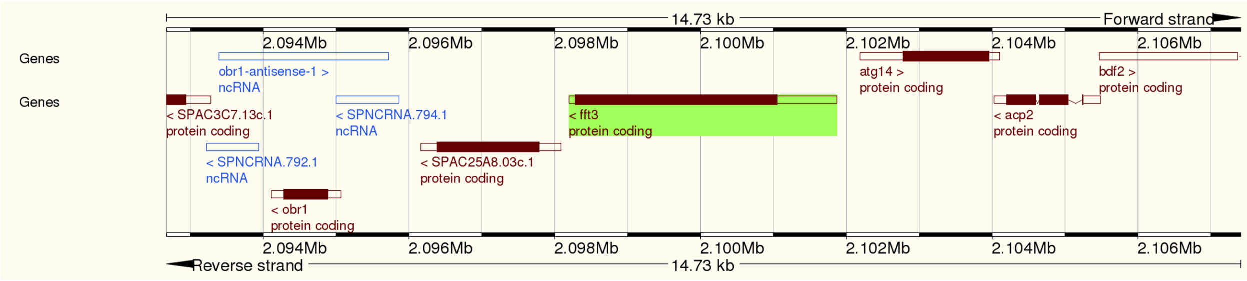

B

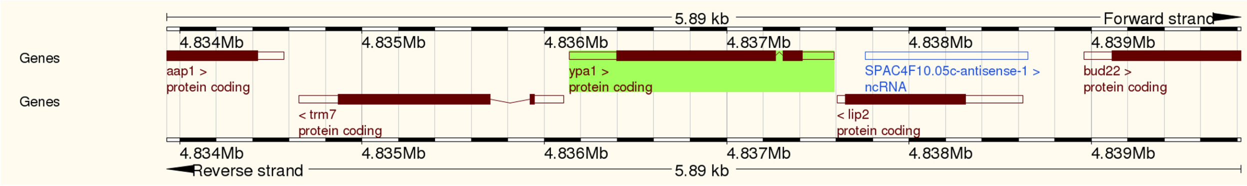

Figure S3 – Ensembl genome browser generated view of the genomic regions surrounding (A) *fft3* or (B) *ypa1*.

Biology Open • Supplementary information

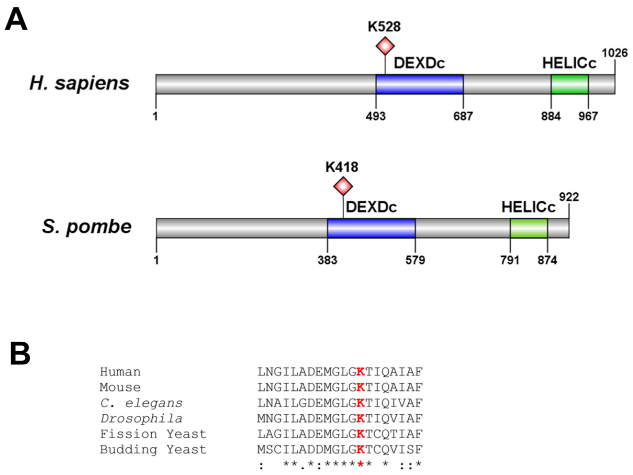

Figure S4 – The ATPase domain of human SMARCAD1 is conserved in fission yeast. (A) Domain structure of human SMARCAD1 compared to its fission yeast ortholog, Fft3p. The conserved helicase domain is shown in green. The conserved lysine residue in the ATPase domain is indicated with a red diamond. (B) A clustalW alignment of the relevant portion of the ATPase domain of Fft3p.

Biology Open • Supplementary information

A

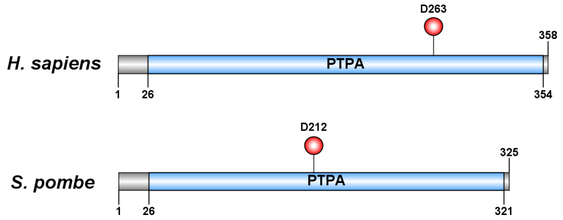

B

```
Human           EPAGSQGVWGLDDFQFLPFIWGSSQLID
Mouse           EPAGSQGVWGLDDFQFLPFIWGSSQLID
C. elegans      EPAGSRGVHAIDDFQFAPFIFGSAQLIG
Drosophila      EPAGSQGVWSLDDFQFVPFIWGSAQLAV
Fission Yeast   EPAGSHGVWGLDDHFFIPYIFGSAQLAD
Budding Yeast   EPAGSHGVWGLDDYHFLPFLFGAFQLTT
                *****:** .:**. * *:::*: **
```

Figure S5 – The PPIase domain of human PTPA is conserved in fission yeast. (A) Domain structure of human PTPA compared to its fission yeast ortholog, Ypa1p. The conserved aspartate residue in the catalytic domain is indicated with a red diamond. (B) A clustalW alignment of the relevant portion of the catalytic domain of Ypa1p.

Biology Open • Supplementary information

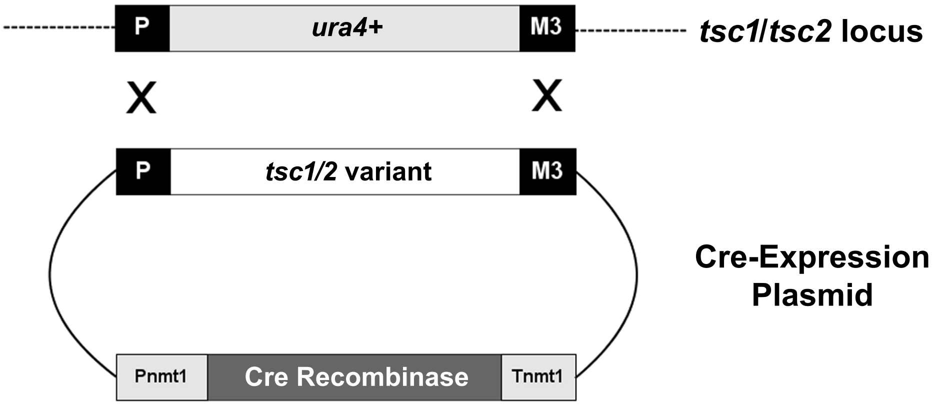

Figure S6 – Schematic describing the recombinase mediated cassette exchange of *tsc1* and *tsc2* variants at the *tsc1/tsc2* loci. P, *loxP* site; M3, *loxM3* site; P_{nmt1}, *nmt1* promoter; T_{nmt1}, *nmt1* terminator.

Biology Open • Supplementary information

SUPPLEMENTARY FILES

Sequence 1: NP_000359.1 **Gene:** *TSC1* **HGNCID:** 12362 **Length:** 1164 **Species:** *H. sapiens*
Sequence 2: NP_593028.1 **Gene:** *tsc1* **PomBaseID:** SPAC22F3.13 **Length:** 899 **Species:** *S. pombe*

Alignment Length: 1033 **Identity:** 187/1033 (18%)
Similarity: 302/1033 (29%) **Gaps:** 480/1033 (46%)

```
Human     41 LVNTLVDYYLETSSQPALHILTTLQEPHDKHLLDRINEYVGKAATRLSILSLLGHVIRLQPSWKH 105
             |||.||.|              .:|.|  |.|.....:......||:.|||::..|||.|....:
pombe    175 LVNVLVHY--------------GIQRP--KELSSCFCHHFLNPPTRIPILSVMVEVIRRQGPRLY 223

Human    106 KLSQAPLLPSLLKCLKMDTDVVVLTTGVLVLITMLPMIPQSGKQHLLDFFDIFGRLS-------- 162
             ::.|......:|||.:.||..::|:..:..::.:|..|..|....|...|.|:.|.|
pombe    224 EIPQTGFYDLVLKCAEFDTSPILLSYALSFILMILSHICNSLDDSLYRLFCIYLRFSMIDPTSGF 288

Human    163 -------SWCLKKPGHVAEVYLVHLHASVYA---------------------------------L 187
                    :|         ||:  |...|.||                                 |
pombe    289 PSSTASGNW---------EVF--HDFMSTYASTTTSQTDSSYNDVHDIVGSSQPDYLESLDYSQL 342

Human    188 FHRLYGMYPCNFVSFLRSH--YSMKENLE---TF-EEVVKPMME-----HVRIHPELVTGSKDHE 241
             |..||.:||.||:.|||..  |:.|.|.:   :| :|::....:     |: .|...:..:.:.|
pombe    343 FSILYALYPINFLEFLRDPKLYASKHNFQIRYSFNQELLSTKSDGLLGRHL-AHSNFLKYTAETE 406

Human    242 L-DPRRWKRLETHDVVIECAKISLDPTEASYEDGYSVSHQISARFPHRSADVTTSPYADTQNSYG 305
             | |..||.||::..||..|                                       ::.|:.|
pombe    407 LTDKSRWTRLDSIAVVALC---------------------------------------NSLNAVG 432

Human    306 CATST--PYSTSRLMLLNMPGQLPQTL--SSPSTRLITEPPQATLWSPSMVCGMTTPPTSPGNVP 366
             .|.|.  |:.          |:||.|.  :|.:|.|:..|                      |..
pombe    433 IAESVMDPFG----------GKLPTTYEETSSATGLLAYP----------------------NES 465

Human    367 PDL-SHPYSKVFGTTAGGKGTPLGTPATSPPPAPLCHSDDYVHISLPQATVTPPRKEERMDSARP 430
             .|: |.|:|                                  ||.||
pombe    466 HDIASEPFS----------------------------------ISWPQ----------------- 479

Human    431 CLHRQHHLLNDRGSEEPPGSKGSVTLSDLPGFLGDLASEEDSIEKDKEEAAISRELSEITTAEAE 495
                             .|...|||
pombe    480 ----------------NPSISGSV----------------------------------------- 487

Human    496 PVVPRGGFDSPFYRDSLPGSQRKTHSAASSSQGASVNPEPLHSSLDKLGPDTPKQAFTPIDLPCG 560
                                     |||.:..:....|.|
pombe    488 ------------------------HSATTFDKAQLSNTE-------------------------- 502

Human    561 SADESPAGDRECQTSLETSIFTPSPCKIPPPTRVGFGSGQPPPYDHLFEVALPKTAHHFVIRKTE 625
                                                      ..||::
pombe    503 -----------------------------------------DSYDNI------------------ 508

Human    626 ELLKKAKGNTEEDGVPSTSPMEVLDRLIQQGADAHSKELNKLPLPSKSVDWTHFGGSPPSDEIRT 690
                  :.|.:..:||.|...::.     ::|:       |.|.|.|:|:       |..:|.||.
pombe    509 -----SHGTSYSEGVSSIHMVKG-----ERGS-------NNLELTSESL-------SSTNDTIRR 549

Human    691 LRDQLLLLHNQLLYERFKRQQHALRNRRLLRKVIKAAALEEH-------NAAMKDQLKLQEKDIQ 748
             |:..||.|.|:|.:|:|.||||.....:|.|:.|...|:|..       |...|.|::|...:|.
pombe    550 LQRDLLFLQNELRFEKFVRQQHLQNIGKLHREHILDMAVESERQKLLLTNKRYKAQIELLNSEID 614

Human    749 MWKVSLQKEQARYNQLQEQRDTMVTKLHSQIRQLQHDREEFYNQSQELQTKLEDCRNMIAELR-- 811
             ..:   .:.||..|    :|.......:::|:.|:.:::.::::..||::.:|...:.:..:|
pombe    615 KHR---SESQAALN----RRVKWENDFNNKIKALREEKKAWKSEESELKSSIESLISQLESIRNS 672

Human    812 -IELKKANNKVCHTELLLSQVSQKLSNSESVQQQMEFLNRQLLVLGEVNELYLEQLQNKHSDTTK 875
              |::..:.|::   ||.|.....||...|.....:....:|:               :..|||:.
pombe    673 QIDIAFSKNQL---ELKLQLYETKLKEYEQHLSCVNISKKQV---------------SSSSDTSF 719

Human    876 EVEMMKAAYRKELEKNRSHVLQQTQRLDTSQKR-ILELESHLAKKDHLLLEQKKYLEDVKLQARG 939
             ....|          :.|.:|..::.:...|:| ::|.|.|..|           ||...|..:.
pombe    720 GNTKM----------DSSMILSNSEAVSDEQERELIESEKHRMK-----------LESENLHLQA 763

Human    940 QLQAAESRYEAQKRITQVFELEILDLYGRLEK---------------DGLLKKLEEEK 982
             .::..:...||   |..|:|.:|.||..||..               |..|.|:.|.|
pombe    764 NIELLKKDLEA---INVVYEAKIFDLEKRLSSEANAPELHNPVNLNYDAQLSKISEIK 818
```

File S1 – Protein sequence alignment of human *TSC1* and *S. pombe tsc1* generated using the DRSC integrative ortholog prediction tool (http://www.flyrnai.org/cgi-bin/DRSC_orthologs.pl). Residues highlighted in red correspond to amino acids altered in clinical cases of TSC.

Sequence 1:XP_011520940.2 **Gene:***TSC2* **HGNCID:**12363 **Length:**1879 **Species:** *H. sapiens*
Sequence 2:NP_592909.1 **Gene:***tsc2* **PomBaseID:**SPAC630.13c **Length:**1339 **Species:** *S. pombe*

Alignment Length: 1773 **Identity:** 341/1773 (19%)
Similarity: 562/1773 (32%) **Gaps:** 684/1773 (39%)

```
Human    174 RALFFKVIKDYPSNE--DLHERLEVFKALTDNGRHITYLEEELADFV-------LQWMD-----V 224
             |..||:.|:..|..:  :|..||:....|||.|..|:.:|..:....:       |||..     .
pombe     97 RHTFFQTIEKDPFQDIAELQLRLKSLSVLTDEGHKISGIENRVGPLLSAWFNQYLQWQSQATELQ 161

Human    225 GLSSEFLLV-----------LVNLVKFNSCYLDE-YIARMVQMICLLCVRTASSVDIEVSLQVLD 277
             |..::..||           ..|||||....:.| .:.::|..:..:|........:...|...|
pombe    162 GKDADSKLVHLLFFKSLFKFSTNLVKFQWFLVPEPQMLQLVNSVVQICNHARLEDVVTEVLMFFD 226

Human    278 AVVCYNCLPAESLPLFIVTLCRT-INVKELCEPCWKLMRNLLGTHLGHSAIYNMCHLMEDRAYME 341
             :::.|:.:|..||...::.||.| |:.....:....::.||:.:.:.:.|..|:.::::......
pombe    227 SMIRYSVIPKASLYDTVLILCSTYISTYSYSKLAQSVIFNLISSPVSNLAFENVFNILQYNRSNV 291

Human    342 DAPLLRGAVFFVGMALWGAHRLYSLRNSPTS--VLPSFYQAMACP---NEVVSYEIVLSITRLIK 401
             :|   :||||..:....:      |..::|...:  .|.|..:....|    ||.|.:||:.:....::
pombe    292 NA--VRGAVRLMRFLM-----LQEVKNDAIASITLSSSIEFTEFPLGFNENVDFEILGTVYLFLR 349

Human    402 --KYRKELQVVAWDILLNIIERLLQ--QLQTLDSPELRTIVHDLLTTVEELCDQNEFHG----SQ 458
               .....|..:.|..:|||:....|  .|:...|.|..:..........:.:.|..:|..    .|
pombe    350 TPSILNRLNFLDWHRILNILMYCSQYLPLKASTSKEAFSKTAAFANIYDRVLDFLDFEALIPLLQ 414

Human    459 ERYFELVERCADQRP------ESSLLNLI-SYRAQSIHPAKDGWIQNLQALMERFFRSESRGAVR 516
             :...:||....|..|      ...||.|. :|..  |.|....|:.||:.|:          |..:
pombe    415 QFQVKLVFFLKDVLPVLKPKIRKKLLRLFETYNL--IFPCNQYWVFNLEFLL---------GIYQ 468

Human    517 IKVLDV----LSFVLLINRQFYEEE-----LINSVVISQLSHIPEDKDHQVRKLATQLLVDLA-- 570
             .|..|:    |.|.|:.:.....:|     |.:..:..:....::.|..|......:|..|:
pombe    469 CKTFDLEDRALLFKLVEDACSVADENSAPILCSKFLFPVIESFSKESDDCVVSPVYNMLFFLSVN 533

Human    571 ------EGCHTHHFNSLLDIIEKVMARSLSPPPELEERDVAAYSASLED---VKTAVLGLLVILQ 626
                   :.|..|.|..|:.....|..|.|:....:.   :..|...|.|   ::..:..:|.||:
pombe    534 FQNPGLKDCIDHIFQQLISDTSSVTVRRLATSTLIR---LFYYYYDLRDAVPIQETLAKMLEILE 595

Human    627 TKLYTLPASHATRVYEMLVSHIQLHYKHSYT-LPIASSIRL-QAFDFLLLLRADSLHRLGLPNKD 689
             |..:..        |..||.....|.::.:.| :.|..:|.| :.|..|              |.|
pombe    596 TPSFPF-------VSRMLCLQFFLRFRANGTSIYICENIDLNEPFKVL-------------NVD 639

Human    690 GVVRFSPYCVCDYMEPERGSEK-----------KTSGPLSPPTGPPGPAPAGPAVRLG----SVP 739
             ..:..:.|.:.|........||              |.|                ..:...|    :.|
pombe    640 SELIPAVYPISDSFVNSATVEKHIWERKENDLIKIS---------------NHSTEYGKDFVTFP 689

Human    740 YSLLFRVLLQCLKQESDWKVLKLVLGRLPESLRYKVLIFTSPCSVDQLCSALCSMLSGPKTLERL 804
             .|.|.|...:.:..||:|.:|..::..|.:.:..:.:..:...:..|...:|.::      .||:
pombe    690 TSSLLRFYRKSMATESNWTILMFMITHLADQISNRSMFIGALEEIYNLLDFMCDIV-----FERV 749

Human    805 ---RGAPEGFSRTDLHLAVVPVLTALISYHNYLDKTKQREMVYCLEQGLIHRCASQCVV---ALS 863
                ...|....:.::.:.::..:..|..||:...:.::.|:|.....|| .:....|.|   :|.
pombe    750 SISAEIPSNIRKANIMIPILQNVQMLFVYHDQFSRAQEDELVSVFFAGL-QKWNEACHVSIHSLM 813

Human    864 ICSVEMPDIIIKALPVLVVKLTHISATASMAVPLLEFLSTLARLPHLYRNFAAEQYASVFAISLP 928
             :|..|:|..|.|.||.::|.|:.:.....::|.:||||.:|||||.|..||....|..:|||:|.
pombe    814 LCCYELPVSIRKQLPAILVTLSRLITKPDLSVHILEFLCSLARLPDLIANFTDADYRQIFAIALK 878

Human    929 Y------TNPSK------------FNQYIVCLAHHVIAMWFIRCRLPFRKDFVPFITKGLRSNVL 975
             |      |..||            ::.|::.||:.|:.:||:..||..||.|||:|.:||:
pombe    879 YIQHRDFTKESKDSNDTESILKNSYSSYVLALAYSVLQIWFLSLRLTERKKFVPWILRGLK---- 939

Human    976 LSFDDTPEKDSFRARSTSLNERPKSLRIARPPKQGLNNSPPVKEFKESSAAEAFRCRSISVSEHV 1040
             |:.:..|
pombe    940 LASEGKP---------------------------------------------------------- 946

Human   1041 VRSRIQTSLTSASLGSADENSVAQADDSLKNLHLELTETCL---DMMARYVFSNFTAVPKRSPVG 1102
                                                 |.:.||    |||.::.:||...    .:...
pombe    947 ------------------------------------LEDLCLVQYDMMQQFCYSNSDI---NNQTS 973
```

Biology Open • Supplementary information

```
Human   1103 EFLLAGGRTKTWLVGNKLVTVTTSVGTGTRSLLGLDSGELQSGPESSSSPGVHVRQTKEAPAKLE 1167
             .|:.:...::||:.||.|.|:..||.:|
pombe    974 TFVDSDVESETWIRGNSLFTINVSVNSG------------------------------------- 1001

Human   1168 SQAGQQVSRGARDRVRSMSGGHGLRVGALDVPASQFLGSATSPGPRTAPAAKPEKASAGTRVPVQ 1232
                                                ||
pombe   1002 -----------------------------------FL---------------------------- 1003

Human   1233 EKTNLAAYVPLLTQGWAEILVRRPTGNTSWLMSLENPLSPFSSDINNMPLQELSNALMAAERFKE 1297
                              |.::|||||.|.:....|..|..|..:.|....:.|:..|:.
pombe   1004 -----------------EAVIRRPTGTTQYTFRNEASLQKFLWEENLTSSKALTRGLLC------ 1045

Human   1298 HRDTALYKSLSVPAASTAKPPPLPRSNTVASFSSLYQSSCQGQLHRSVSWAGIASQREAVGCRAP 1362
                                        |.:||.|.:
pombe   1046 ---------------------------TPSSFVSHF----------------------------- 1054

Human   1363 LCLIDSAVVMEEGSPGEVPVLVEPPGLEDVEAALGMDRRTDAYSRSSSVSSQEEKSLHAEELVGR 1427

pombe   1055 ----------------------------------------------------------------- 1054

Human   1428 GIPIERVVSSEGGRPSVDLSFQPSQPLSKSSSSPELQTLQDILGDPGDKADVGRLSPEVKARSQS 1492

pombe   1055 ----------------------------------------------------------------- 1054

Human   1493 GTLDGESAAWSASGEDSRGQPEGPLPSSSPRSPSGLRPRGYTISDSAPSRRGKRVERDALKSRAT 1557
                                                |.|.|
pombe   1055 -----------------------------------LDPHG------------------------- 1059

Human   1558 ASNAEKVPGINPSFVFLQLYHSPFFGDESNKPILLPNESQSFERSVQLLDQIPSYDTHKIAVLYV 1622
                             :.||         |:|:|||:...|..|::.:.|:||..::.|..::||
pombe   1060 ----------------ISLY---------NQPLLLPSNDDSVRRAISVFDRIPVIESLKAGLVYV 1099

Human   1623 GEGQSNSELAILSNEHGSYRYTEFLTGLGRLIELKDCQPDKVYLGGLDVCGE-DGQFTYCWHDDI 1686
             |. |...|..||:|.:.|..:..||.|||.|.|||..|  ||:.||||...: ||.|.|||.|.:
pombe   1100 GY-QQRREADILANTNPSEDFLTFLNGLGTLFELKTDQ--KVFAGGLDRENDIDGAFAYCWKDKV 1161

Human   1687 MQAVFHIATLMPTKDVDKHRCD-KKRHLGNDFVSIVYNDSGEDFKLGTIKGQFNFVHVIVTPLDY 1750
             .|.|||..|:|||.......|. ||||:||||:|::|:||.::...||..|||||::::||...
pombe   1162 TQMVFHCTTMMPTNIEHDPGCTLKKRHIGNDFVTIIFNESGLEYDFDTIPSQFNFVNIVITPESE 1226

Human   1751 ECNLVSLQCR-KDMEGL----VDTSV---AKIVSDRNLPFVARQMALHANMASQVHHSRSNPTDI 1807
             .......|.: ..::.|    :|.|:   .||||...||.:.|.:.|:|.:.|.::|..:..
pombe   1227 SIRRTGRQIKFYKVKALTKYDIDFSLFRRYKIVSSDALPAIVRDVTLNAAVFSHIYHRSAGD--- 1288

Human   1808 YPSKWIARLRHIKRLRQR 1825
             |...|..|||.:||||::
pombe   1289 YVHIWAERLRQLKRLREK 1306
```

File S2 – Protein sequence alignment of human *TSC2* and *S. pombe tsc2* generated using the DRSC integrative ortholog prediction tool (http://www.flyrnai.org/cgi-bin/DRSC_orthologs.pl). Residues highlighted in red correspond to amino acids altered in clinical cases of TSC.

Biology Open • Supplementary information
